# A Higher Abundance of O-Linked Glycans Confers a Selective Advantage to High Fertile Buffalo Spermatozoa for Immune-Evasion From Neutrophils

**DOI:** 10.3389/fimmu.2020.01928

**Published:** 2020-08-28

**Authors:** Vipul Batra, Komal Dagar, Samiksha Nayak, Arumugam Kumaresan, Rakesh Kumar, Tirtha K. Datta

**Affiliations:** ^1^Animal Genomics Laboratory, Animal Biotechnology Centre, National Dairy Research Institute, Karnal, India; ^2^Theriogenelogy Laboratory, SRS of National Dairy Research Institute, Bengaluru, India

**Keywords:** sperm, sugars, lectins, fertility, neutrophils, phagocytosis, NETosis

## Abstract

The glycans on the plasma membrane of cells manifest as the glycocalyx, which serves as an information-rich frontier that is directly in contact with its immediate milieu. The glycoconjugates (GCs) that adorn most of the mammalian cells are also abundant in gametes, especially the spermatozoa where they perform unique reproduction-specific functions e.g., inter-cellular recognition and communication. This study aimed to implicate the sperm glycosylation pattern as one of the factors responsible for low conception rates observed in buffalo bulls. We hypothesized that a differential abundance of glycans exists on the spermatozoa from bulls of contrasting fertilizing abilities endowing them with differential immune evasion abilities. Therefore, we investigated the role of glycan abundance in the phagocytosis and NETosis rates exhibited by female neutrophils (PMNs) upon exposure to such spermatozoa. Our results indicated that the spermatozoa from high fertile (HF) bulls possessed a higher abundance of O-linked glycans e.g., galactosyl (β-1,3)N-acetylgalactosamine and N-linked glycans like [GlcNAc]1-3, N-acetylglucosamine than the low fertile (LF) bull spermatozoa. This differential glycomic endowment appeared to affect the spermiophagy and NETosis rates exhibited by the female neutrophil cells (PMNs). The mean percentage of phagocytizing PMNs was significantly different (*P* < 0.0001) for HF and LF bulls, 28.44 and 59.59%, respectively. Furthermore, any introduced perturbations in the inherent sperm glycan arrangements promoted phagocytosis by PMNs. For example, after *in vitro* capacitation the mean phagocytosis rate (MPR) rate in spermatozoa from HF bulls significantly increased to 66.49% (*P* < 0.01). Likewise, the MPR increased to 70.63% (*p* < 0.01) after O-glycosidase & α2-3,6,8,9 Neuraminidase A treatment of spermatozoa from HF bulls. Moreover, the percentage of PMNs forming neutrophil extracellular traps (NETs) was significantly higher, 41.47% when exposed to spermatozoa from LF bulls *vis-à-vis* the spermatozoa from HF bulls, 15.46% (*P* < 0.0001). This is a pioneer report specifically demonstrating the role of O-linked glycans in the immune responses mounted against spermatozoa. Nevertheless, further studies are warranted to provide the measures to diagnose the sub-fertile phenotype thus preventing the losses incurred by incorrect selection of morphologically normal sperm in the AI/IVF reproduction techniques.

## Introduction

The mammalian spermatozoon is a highly specialized epidermal cell enclosed in a thick glycocalyx (20–60 nm) that contains an array of structurally and functionally diverse glycans. These glycans mediate and facilitate numerous molecular and cellular interactions occurring in diverse immunological, physiological and reproductive processes (1–3). The process of establishment of the sperm glycocalyx begins with the *de novo* synthesis of the glycoconjugates (GCs) in the ER/Golgi of the nascent spermatozoa and is continuously regulated in a Spatio-temporal fashion across the male reproductive tract (MRT) (4, 5). The terminally differentiated spermatozoa which are relatively quiescent in information pathways, exit from the testis and enter the epididymal lumen. In the epididymis, multiple sperm surface remodeling (SSR) events drastically transform the sperm-surface e.g., by tailoring the glycocalyx structure and changing its lipid and protein profiles thus making the spermatozoa capable of survival and performance in the FRT (6). These events which extensively affect the arrangement, composition and structure of sperm glycocalyx occur majorly due to (i) adsorption, insertion, modification and binding of a variety of (glyco)proteins (ii) the glycan modifying enzymes e.g., glycosyltransferases and glycosidases and (iii) intercalation of the glycoconjugates and microvesicles, known as epididymosomes (2, 7–12). The associated changes in sperm-surface glycan distribution during the epididymal transit have recently been reported (13). The glycosylated proteins acquired by the spermatozoa during their transit in the epididymis and pre-ejaculatory ducts are known to play crucial roles in successful fertilization (14–18). These radical changes in the sperm-surface are thus crucial not merely for surmounting the various physio-chemical and immunological barriers in the FRT but also for the molecular mechanisms responsible for sperm-egg interactions (14, 19). The epididymis, therefore, acts as a site of high extracellular glycosyl metabolism involving somatic modifications of the glycocalyx (transglycosylation).

The glycans present on the sperm surface assist the sperm in evading the immune response, mounted against these allogenic cells in response to insemination (3, 14, 20, 21). In the uterus, the entry of the spermatozoa triggers a massive influx of leukocytes, mainly the neutrophils (PMNs) into its lumen (22). The neutrophils adopt two major mechanisms to eliminate sperm from the uterus, either by direct binding and phagocytosis or through the formation of neutrophils extracellular traps apart from other effector mechanisms (23). The sperm glycocalyx is the primary interface directly in contact with the immunologic milieu of the FRT. It has been demonstrated that the FRT is under hormonal control not only to promote fertility but also to concomitantly provide protection from the ascending infectious microorganisms (24–26). However, it is still not clear which sperm-surface molecules are actually recognized by the female PMNs and if the interactions between these two cells are merely random. Nevertheless, the glycan-mediated interactions appear to be the likely mechanism since such interactions have long been described individually for the spermatozoa (27, 28) and the PMNs (29). Interestingly, the recently described sperm-PMN interactions implicated sialic acids on the sperm surface as the facilitators of sperm-survival in the FRT in the face of innate immunity (5, 11). The implications of the classic inflammatory response after insemination, the leukocyte reaction, are especially important in farm animals like buffalo which are routinely subject to artificial insemination (AI). This is because the bovine semen used for AI is processed after collecting the ejaculate. This processing dilutes the seminal plasma components (SPCs) that otherwise help in reducing the neutrophil binding to the spermatozoa (23, 30). The interference with the sperm transport in FRT, associated with the processed semen used in AI, has been demonstrated to reduce the fertility rates because neutrophils are deemed counter-productive to the survival of the sperm in the FRT (23, 30–32). Although the maturity and integrity of the sperm glycocalyx appear to be crucial to male fertility (1, 33) the role of sperm associated glyco-topography (SpAGT) in immune-evasion is not well-understood. Whether it allows some spermatozoa to survive the mounted immune response in the FRT, thus regulating fertility is yet to be elucidated (3, 34–36).

The glycans associated with the sperm plasma membrane appear to be embellished in a manner to convey a vast amount of information pertaining not only to intra- and intercellular communication required for the co-operation and survival in the FRT but also for successful fertilization of the ovum in the ampullary-isthmus junction (37). For example, the identity of a cell to be distinguished as “self” is displayed by the complex glycocalyx of major cell types. The sperm-surface associated glycans terminating in sialic-acid moieties reportedly function as self-associated molecular patterns, SAMPs which interact with the inhibitory receptors on the immune cells presumably facilitating sperm survival in the FRT (11, 38–42). The sialic-acid binding Ig-like lectins (siglecs) on the leukocytes, which are the cognate endogenous binding partners of sialic-acid and certain C-type lectin receptors present on dendritic cells have been reported to be the SAMP receptors that can inhibit and/or modulate immune responses (43–45). The sialic acids are known to alter not only the affinity of a molecule for its ligand but also can change its cognate binding partner (46). Thus, any changes in glycosylation are expected to impact the function and binding interactions of the immune proteins. Besides, the sperm-oocyte interaction in bovine has been demonstrated to involve α-2,3 sialic acid on the oocyte zona pellucida and its cognate siglec on the bull sperm-surface indicating their role in cell-cell recognition (47). The carbohydrate-binding molecules in the FRT e.g., Toll-like receptors (TLRs) and lectins function as pattern recognition receptors (PRRs) and identify the unique spatial arrangement of the GCs such as pathogen-associated molecular patterns, PAMPs, danger-associated molecular patterns, DAMPs and probably the SAMPs too (48–52). The PRRs in the immune cells of the FRT appear to interpret the sperm glycome because a considerable proportion of spermatozoa are destroyed in the uterus by polymorphonuclear neutrophils (PMNs). Yet, enough number of spermatozoa survives to reach the oviduct and one or few eventually fertilize the egg (53–56). The exact molecular mechanisms implicated in this phenomenon of “cryptic female choice” are rather controversial and remain to be elucidated in detail (4, 7, 8, 11, 57–60). The formulation of the complete molecular glycan cartograph depicting the spatial arrangement of the GCs in the SpAGT would be crucial to decode the information about immune-evasion coded in the sperm glycocalyx.

Considering the massive diversity originating from the monomer units, their isomers, linkages, and terminal modifications, the information storage capabilities of the glycocalyx appear no less than that of the genome (61–64). Since the last decade, there has been a spurt in the efforts to define and characterize the vast array of glycans associated with the sperm, nevertheless, very few studies reported the molecular functions of the glycans especially in the “cryptic female choice” of certain selected spermatozoa by the FRT (4, 65–68). The specific-sugar binding proteins, the lectins are well suited for distinguishing the GCs because of their complex specificities, ability to differentiate between the glycoforms, branching, linkage, spacing and multivalency (3, 69–72). Lectins have been used to visualize cell surface structures, detect and analyze the expression and spatial-distribution, alteration of the GCs on the sperm surface and also to evaluate the capacitation and acrosome reaction which change the sperm surface drastically (35, 56, 73–75). The precise structures, composition, valence, linkage and attachment sites of the sugar units need to be determined not only to understand the role of glycosylation in immune-evasion but also to harvest the pertaining information contained in the glycan code which currently, however, is largely unknown. This study was designed to interpret the role of the differential abundance of the glycan moieties present on the sperm surface in the neutrophil-sperm interactions by observing the occurrence of phagocytosis and NETosis in high and low fertile buffalo bull spermatozoa. We hypothesized that any aberrance or change in abundance of sperm surface glycans would perturb the normal glycocalyx structure by molecular alterations. This would disrupt its downstream communication flow leading to the increased PMN phagocytosis and NETosis of the buffalo spermatozoa.

## Methods

### Chemicals and Materials

All chemicals, media and reagents were procured from Sigma Aldrich Chemical Co. Ltd, (USA) unless stated otherwise. All plasticware were procured from Nunc Inc. (Thermo Scientific, USA). Fetal bovine serum (FBS) was obtained from Hyclone, Canada. The FITC-conjugated lectins ABL (*Agaricus bisporus*), MAL II (*Maackia amurensis)* and JAC (*Jacalin*) were obtained from EY Laboratories Inc., CA, U.S.A. The de-glycosylation enzymes were procured from NEB Inc., MA, U.S.A.

### Lectin Cytochemistry (Lectin Binding Assay) –Fluorescence Microscopy

#### Semen Sample Collection From Bulls of Contrasting Fertility and Its Processing

Three high and three low fertile Murrah bulls were selected based on their conception rate (CR) data available at the Artificial Breeding Research Center (ABRC), NDRI, India. Because of the low CRs reported in Buffalo, the bulls with overall CR < 30% were classified as LF while bulls with CR > 45% were categorized as HF bulls. Their frozen semen straws were procured from the center (Supplementary Methods: Table 1). The straws were thawed by immersing them in a water bath at 38°C for 30 s and the contents were collected into 15 ml centrifugation tube containing 2 ml working non-capacitating medium, NCM (1 mM 60% Na-lactate and 0.98 mM Na-Pyruvate in 2X filtered stock mixed with an equal amount of Milli-Q water) (Supplementary Methods: Table 2). The spermatozoa were separated from the semen by centrifuging at 280 x g for 6 min. thrice in working NCM. This removes the seminal plasma components (SPCs) and separates the spermatozoa from the semen. The supernatant was discarded each time and the sperm pellet obtained after the final wash was subject to the swim-up technique. The obtained fraction containing the motile spermatozoa was suspended in 250 μl working-NCM.

#### Standardization of Lectin Concentration for Sperm Surface Binding

A panel of six lectins *viz. Agaricus bisporus* lectin (ABL), *Jacalin* (JAC), *Maackia amurensis* **(**MAL II), *Lycopersicon esculentum* (LEL), *Lens culinaris* agglutinin (LCA), and Peanut agglutinin (PNA) was used to detect the residues of diverse glycan moieties present on the buffalo sperm surface. The lectin concentrations that provided the maximum signal to background noise values preferably near the saturation point of the signals (point of inflection) were subsequently used throughout this study (Supplementary Methods: Table 3).

#### Lectin Binding Assay and Quantification of the Fluorescence Signal

The post-swim-up spermatozoa (10 × 10^6^) in working-NCM (40 μl) were incubated with 15 μl of different lectins at their standardized concentrations in 1.5 ml microcentrifuge tubes (MCTs) at 37°C for 15 min under darkness. The samples were then washed with NCM and smeared on cleaned glass slides. One drop of mounting medium, 1, 4-Diazabicyclo[2.2.2]octane, Dabco® 33-LV solution was placed on each air-dried smear before putting a coverslip on it. The slides were observed under an Olympus BX-51 fluorescence microscope at 1000X magnification using Blue-filter. The micrographs were afterwards used for fluorescence quantification analysis using the ImageJ software. The fluorescent regions were identified and quantified for a minimum of 100 spermatozoa in at least 10 fields covering the entire slide (*n* = 3 biological replicates × 2 technical replicates). The region of interest (ROI) on the individual spermatozoon was selected according to the lectin binding pattern e.g., the head region was considered as the ROI for LCA, LEL and PNA, whereas the mid-piece was the ROI for MALII. The whole sperm periphery was considered for quantification of the fluorescence signal produced upon ABL and JAC binding. The fluorescence intensities were quantified at random from the micrographs that were captured using similar acquisition settings (exposure time, magnification etc.). The raw integrated density values for the chosen ROIs were later normalized and these values were used to obtain the mean fluorescent intensity (MFI) values used in subsequent experiments.

#### *In vitro* Capacitation of Buffalo Bull Spermatozoa and Signal (FITC-Lectin Binding) Quantification in Capacitated Sperm

The *in vitro* capacitation of the buffalo bull spermatozoa was induced by suspending the spermatozoa in the capacitating medium (working-NCM supplemented with 6 mg/ml bovine serum albumin (BSA), 2 mM CaCl_2_.2H_2_O and 10 mM NaHCO_3_) for 6 h in a 5% CO_2_ incubator at 37°C after washing twice in the capacitating medium (CM) containing 1 and 3 mg/ml BSA, consecutively. The sperm capacitation was confirmed by chlortetracycline (CTC) staining. Briefly, 100 × 10^6^ post-swim-up spermatozoa (40 μl) were incubated with 40 μl of CTC working stain in 1.5 ml MCTs at 37°C for 20 min in dark. Later 3.2 μl of 4% paraformaldehyde (PFA) was added and incubated for 5 min. The samples were then washed, smeared on glass slides onto which one drop of mounting medium, Dabco® 33-LV was placed and were observed at 1000X magnification under Olympus BX-51 fluorescence microscope using UV-filter. The capacitated spermatozoa were then subject to lectin binding assays. The mean fluorescence intensity (MFI) values of the individual, capacitated spermatozoa from HF and LF bulls was estimated using ImageJ. The fluorescence signals were quantified after capacitation of HF and LF bull spermatozoa, as already explained.

#### De-glycosylation of High Fertile (HF) Buffalo Spermatozoa and Its Lectin Cytochemistry

##### Standardization of enzyme incubation time and membrane integrity test

The optimum time of the incubation of spermatozoa with the de-glycosylation enzymes (alpha-2,3,4,6,8,9 neuraminidase and O-glycosidase and PNGase F) was decided based on the reduction in the fluorescence signal intensity collected hourly at four time-points in addition to the 0 h control group. The final selected incubation-times that didn't affect the sperm viability significantly while removing the sugars efficiently were subsequently used throughout this study (Supplementary Methods: Section S.1).

##### De-glycosylated buffalo sperm – lectin binding assay

The buffalo bull spermatozoa (10 × 10^6^/ml) were incubated with PNGase F (5 × 10^3^ U/ μl) for 1 h in a 5% CO_2_ incubator at 37°C to remove the N- linked glycans. While, for the complete removal of O-linked glycans, the spermatozoa (10 × 10^6^/ml) were initially incubated with alpha-2,3,4,6,8,9 Neuraminidase (20 U/μl) to remove the terminal sialic acid moieties from its glycocalyx. Later, the same sample was incubated with 2 μl of O-glycosidase (40 × 10^3^ U/μl) in 80 μl of NCM for 2 h in 5% CO_2_ incubator at 37°C to remove the complete core of O-linked oligosaccharides. The image acquisition and subsequently the lectin fluorescence quantification was performed for the deglycosylated and the control spermatozoa (no enzymes) samples, as explained earlier.

### Lectin-Binding Assay (Validation): Flow Cytometry Analysis

To validate the findings of fluorescence microscopy and to quantify the lectin binding in high throughput and less subjective manner, flow cytometry was performed on (i) spermatozoa from HF and LF bulls (ii) capacitated and non-capacitated spermatozoa from HF bulls (iii) intact(control) and de-glycosylated spermatozoa from HF bulls. The validation of differential abundance and removal of sugars using deglycosylation enzymes was done using three lectins *viz*. ABL, JAC and LEL. However, all the six lectins including MAL II, LCA and PNA were used for validating the re-organization of glycans after *in vitro* capacitation of the buffalo bulls' spermatozoa. Briefly, the spermatozoa were washed, as described earlier and their concentration was adjusted to 3 × 10^6^ sperm/ml. Samples were then incubated with lectins for 10 min at 38.6°C under an atmosphere of 5% CO_2_ in dark, before flow cytometry which was performed with a standard bench-top BD Accuri C6 flow cytometer (Becton Dickinson Biosciences, Ann Arbor, MI, USA, with BD Accuri C6 software v.1.0.27.1). The machine was calibrated daily according to the manufacturer's recommendations with 8 and 6 peak calibration beads, and the QC was performed every second day using BD CS&T RUO beads. The 488-nm laser was used for the excitation of FITC, and its emission was filtered using a 533/30 bandpass filter. The filtered emissions were detected by photomultiplier tubes. A threshold of 80,000 in the forward scatter (FSC) signal was applied to remove the electrical noise and very small events. The samples were collected at the default low flow rate (14 μl/min). For each sample, 20,000 individual events were acquired. The cell population of events in FSC-SSC dot plots was identified and used for the data analysis. No compensation or voltage gain settings were applicable. Further analysis was done using BD FlowJo software v10.6.1 and the MIFlowCyt (76) guidelines were followed to the maximum extent possible.

### Sperm-Neutrophil (PMN) Challenge

#### Preparation of Pure Neutrophil (PMN) Cells From Blood

Blood was isolated from the jugular vein of healthy female Murrah buffaloes in the EDTA coated vacutainers. The PMN cells (neutrophils) were isolated from the blood using Polymorphprep (Cosmo Bio USA, CA, USA) as per manufacturer's instructions (Supplementary Methods: Section S.2). The viability of the PMN cells was determined by the trypan blue exclusion test. The isolated PMNs were further characterized by their “hallmark” nuclear morphology and monoclonal antibody CH138a against the lineage-specific marker CD11c (Supplementary Methods: Sections S.2.1, S.2.2).

##### Phagocytosis assay for capacitated and non-capacitated high fertile (HF), low fertile (LF) bull spermatozoa and for the de-glycosylated high fertile (HF) bull spermatozoa

The non-capacitated and capacitated spermatozoa (40 × 10^6^) from HF and LF bulls or the de-glycosylated sperm from HF bulls in NCM were incubated with the isolated PMNs (10 × 10^6^) in a 96-well polystyrene microtiter plate. The plate was then placed in an orbital shaker with mild swirling for 90 min. After incubation, an equal volume of heparin (40 mg/ml in working-NCM) was added to avoid agglutination of the neutrophil cells (PMNs). The sample at this stage was also used for electron microscopy experiments (explained later). However, for the phagocytosis assay, 75 μl of this sample was fixed in 25 μl of 2% (v/v) glutaraldehyde, a drop (20 μl) of which was examined under an Olympus BX-51 microscope after 20 min. of fixation. One hundred PMNs, in triplicates, were counted in minimum 10 fields and the percentage of PMNs which were either phagocytizing or interacting with the spermatozoa was calculated.

### NETosis Assay

As mentioned above, the spermatozoa and the isolated PMNs were incubated, nonetheless, for 3 h for NETosis experiments in an orbital shaker with mild swirling. Subsequently, 75 μl of this sample was fixed in 25 μl of 2% (v/v) glutaraldehyde, a drop (20 μl) of which was examined under an Olympus BX-51 microscope after 20 min. of fixation. For immunofluorescence (ICC), another smear was prepared, albeit on a poly-L-lysine coated slide and the cells were fixed after 5 min by adding 4% PFA (in PBS) for 20 min at RT. The cells were then washed with PBS thrice and permeabilized by adding PBST (0.2% Triton in PBS). The cells were again washed thrice with PBS and incubated in the blocking buffer (2% BSA in PBST) for 30 min. The cells were then incubated with primary monoclonal antibody anti-myeloperoxidase (anti-mouse, Cloud- clone corp.USA, 1:200) in blocking buffer for 1 h at RT. The samples were then washed twice with PBST and incubated with FITC-conjugated anti-mouse secondary antibody (1:500) in dark for an hour. Thereafter, the cells were washed twice with PBST, slide kept for air-drying and examined under an Olympus BX-51 microscope. One hundred PMNs were counted in a minimum of 10 fields in duplicates.

#### Scanning Electron Microscopy

The buffalo sperm samples used for phagocytosis were fixed in a mixture of 2% paraformaldehyde and 2.5% glutaraldehyde in 0.1 M phosphate buffer (pH 7.4) for 12 h at 4°C. The samples were then centrifuged at 1,000 rpm for 10 min at RT and the supernatant was discarded. The pellet was suspended in 0.1 M phosphate buffer (pH 7.4) and again centrifuged at 1,000 rpm for 5 min. It was then resuspended in the buffer and a drop of it was spread on a coverslip. The samples were air-dried, sputter-coated (SCD 050 Super Cool Sputter System; Baltec Technology, Liechtenstein) with colloidal gold and observed under an EVO 18 scanning electron microscope (Carl Zeiss) at an operating voltage 20 kV. The images were digitally acquired by using the SmartSEMsoftware attached to the microscope at 5000-10000X magnification.

#### Expression Analysis of a Crucial NETosis Gene, Myeloperoxidase by RT-qPCR

The RNA extraction from the PMNs incubated with spermatozoa and the unchallenged PMN groups (control), preparation of their cDNA and primer design were done using standard procedures (Supplementary Methods: Sections S.3, S.3.1, S.3.2). The relative quantification of a crucial NETosis gene, the MPO, was done on a BioRad CFX96 RT-qPCR using Maxima SYBR green master mix (Fermentas, USA) in a 10 μl reaction volume. The amplification was carried out at 95°C for 5 min, followed by 40 cycles consisting of denaturation at 95°C for 10 s, annealing at 63°C for 15 s and extension at 72°C for 15 s. The melting curve analysis was performed for 10 s at 95°C and then 60 s each at 0.5°C increments between 65 and 95°C. A no-template control (NTC) was run in each plate to confirm the absence of gDNA contamination. The relative expression of the MPO gene was calculated using the ΔΔC_t_ (cycle threshold) method and compared with the geometric average of two reference genes, Ribosomal Protein S18 (RPS18) and Eukaryotic elongation factor 2 (eEF2). The control group was taken as the calibrator. The differential expression levels among the control and challenged groups were analyzed by ANOVA and Tukey's *post-hoc* test as implemented in the GraphPad Prism 7.0 (GraphPad Software, La Jolla California USA) software.

### Data Analysis

The quantitative differences in the MFIs produced upon lectin-binding between the HF and LF bulls were analyzed by an unpaired two-tailed *t*-test and a *P*-value < 0.05 was considered statistically significant. The differential MFI levels between capacitated and non-capacitated spermatozoa were analyzed by a paired two-tailed *t*-test. Additionally, a two-way ANOVA followed by Tukey *post-hoc* multiple comparison test was performed to determine the effect of capacitation, fertility status and their interaction on the MFI signal produced for each lectin. The effect of de-glycosylation on MFI of lectins in HF bulls was analyzed by a paired two-tailed *t*-test. The differential rate of phagocytosis by the PMNs (neutrophils) incubated with either HF or LF sperm was analyzed by an unpaired two-tailed *t*-test. The differential rate of phagocytosis in PMNs subjected to capacitated and non-capacitated spermatozoa was, however, analyzed by a paired *t*-test. The differential rate of phagocytosis in PMNs incubated with either HF or PNGase F/O-glycosidase (along with Neuraminidase) treated HF spermatozoa were analyzed by one-way ANOVA followed by Tukey's *post-hoc* multiple comparison test. The differential rate of NETosis in HF and LF bulls was analyzed by an unpaired two-tailed *t*-test. All the tests were implemented in the GraphPad Prism 7.0 software (for Windows, GraphPad Software, La Jolla California USA).

## Results

### The Localization Pattern of Sugars on the Buffalo Spermatozoa Surface

**Figure 1:**
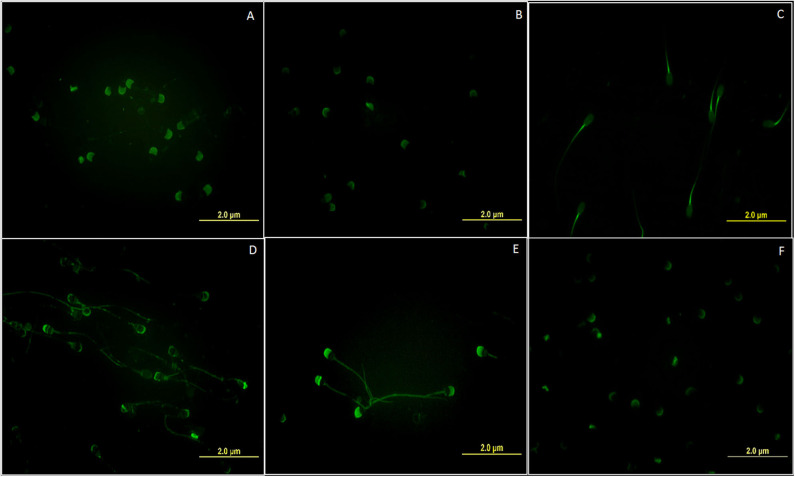
Fluorescent micrographs (magnification 1000x) depicting the differential spatial distribution of the cognate glycan moieties for the six FITC labeled lectins **(A)** LEL **(B)** LCA **(C)** MAL-II **(D)** ABL **(E)** JAC and **(F)** PNA on the surface of the buffalo spermatozoa. All images were captured using the same acquisition settings.

To identify the localization patterns of sugars on the buffalo spermatozoa surface, a sperm-lectin binding assay also known as lectin cytochemistry (LCC) was performed with the optimized concentrations (Table 1 and Supplementary Results: Figure 1) of the six selected lectins. These optimized concentrations were used for other analyses throughout the study.

**Table 1:** Optimized concentrations of the lectins.

**Lectin**	**Optimized concentration**	**Major sugar recognized**
LEL (*Lycopericon esculentum* lectin)	25 μg/ml	[GlcNAc]1-3, N-acetylglucosamine
ABL (*Agaricus bisporus* lectin)	50 μg/ml	galactosyl (β-1,3) N-acetylgalactosamine
JAC (Jacalin)	50 μg/ml	Mono or di-sialylated of T-antigen
MAL II (*Maackia amurensis* lectin II)	20 μg/ml	α-2,3 linked Sialicacid
LCA (*Lens culinaris* agglutinin)	50 μg/ml	Mannose and Glucose moieties
PNA *(Peanut* agglutinin)	50 μg/ml	Asialylatedgalactosyl (β-1,3) N-acetylgalactosamine

Each of the six selected lectins was found to bind a specific, however, different region on the buffalo spermatozoa, thus exhibiting a spatial variation (differential distribution) in their binding patterns. For this study, the sperm populations homogenous in morphology, motility and viability from the HF and LF bulls, were used to rule out the possibility that the observed spatial differences were due to sperm heterogeneity between the two fertility groups (Supplementary Results: Table 1). The lectin from *Lycopersicon esculentum* (LEL), which is specific for [GlcNAc]1-3, N-acetylglucosamine moiety produced a maximum fluorescence signal on the acrosomal and post-acrosomal regions upon binding the head region of the buffalo spermatozoa (Figure 1A). The *Lens culinaris* agglutinin (LCA) which specific to mannose and glucose monosaccharides (Figure 1B) also emitted a fluorescence signal on the acrosomal region, however, with a much weaker intensity. Nevertheless, the *Maackia amurensis* lectin (MALII) which has an affinity for α-2, 3 linked sialic acids was found to preferentially bind only the mid-piece of the buffalo spermatozoa (Figure 1C). The *Agaricus bisporus* lectin (ABL), which binds specifically to the O-linked glycans like the galactosyl (β-1,3)N-acetylgalactosamine moieties (3), generated a patchy fluorescence along the periphery upon binding the buffalo sperm with a slightly strong fluorescence signal intensity on the outer acrosomal region (Figure 1D). The lectin from *Jacalin* (JAC) which recognizes the galactose moiety also binds the O-linked glycans, preferentially with the structure galactosyl (β-1, 3) N-acetylgalactosamine (3). It was found to bind uniformly over the whole buffalo sperm-surface of buffalo however, with a marked rise in signal intensity in proximal regions of the spermatozoa head (Figure 1E). Interestingly, JAC also produced an ABL-like patchy fluorescence pattern in a subset of buffalo spermatozoa, nevertheless, this fluorescent signal was not only greater in intensity but also covered a wider area compared to the ABL signal (Supplementary Results: Figure 10). The lectin from *Arachis hypogea*, the peanut agglutinin (PNA) which binds only to the asialylated galactosyl (β-1, 3) N-acetylgalactosamine moiety produced fluorescence only on the outer acrosome membrane upon binding the buffalo sperm (Figure 1F). Therefore, it appears that the cognate glycans for these lectins exhibit differential distribution pattern and occupy specific regions in the different segments of the buffalo spermatozoa.

### Differential Lectin Binding Between the Bulls of Contrasting Fertilizing Abilities

**Figure 2:**
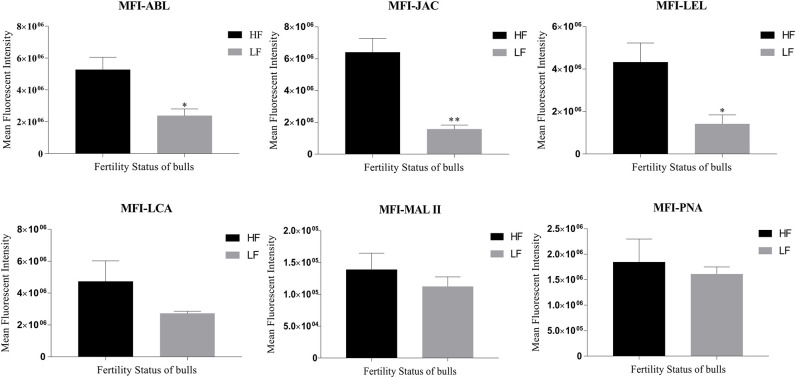
Bar graphs of the mean fluorescent intensity (MFI) values obtained after quantification of the fluorescent signal produced during LCC (lectin cytochemistry) experiments using ImageJ. The signal was produced upon binding of the six FITC-conjugated lectins *viz*. ABL, JAC, LEL, LCA, MAL II, and PNA on the buffalo spermatozoa from bulls (*n* = 3) of contrasting fertilizing abilities. The difference in MFI values was assessed by an unpaired two-tailed *t*-test. HF, high fertile; LF, low fertile bull spermatozoa. *P < 0.05 and **P < 0.01.

The LCC and fluorescent signal quantification were performed using the six lectins on the spermatozoa from buffalo bulls of high and low fertility which were selected based on their contrasting conception rates (CRs). Approximately 10–12% of the total buffalo sperm population was found to be unstained, as inferred from the comparison of fluorescent micrographs with the bright-field micrographs. This unstained sperm-population was excluded from the fluorescence quantification analyses. Three out of the six selected lectins *viz*. ABL, JAC and LEL were found to display significantly differential fluorescent intensities between the spermatozoa from HF and LF bulls (Figure 2 and Supplementary Results: Figure 2). For example, the average of the mean fluorescence intensity (MFI) values produced upon ABL binding was almost half in the spermatozoa from LF *vis-à-vis* the MFI in HF bull spermatozoa. Precisely, it was 54.77 percentage points lower (*p* < 0.05) in the spermatozoa from the LF bulls (Figure 2). In the case of JAC, both the already explained fluorescence patterns were considered for quantification, nonetheless, the spermatozoa were chosen at random. The MFI produced upon JAC binding to the spermatozoa from LF bulls was significantly lower (*p* < 0.01) by an astounding 75.50 percentage points in comparison to the spermatozoa from the HF bulls. A similar trend was observed for LEL binding wherein the average MFI, for the spermatozoa from LF bulls, was 67.26 percentage points lesser (*p* < 0.05) than the average MFI value for the HF bull spermatozoa (Figure 2). The average MFIs for LCA, MALII and PNA upon binding the LF buffalo sperm were 42.50, 19.08, and 12.72 percentage points lower, respectively than the HF buffalo sperm, however, the differences were found to be non-significant (Figure 2 and Supplementary Results: Figure 2).

**Figure 3:**
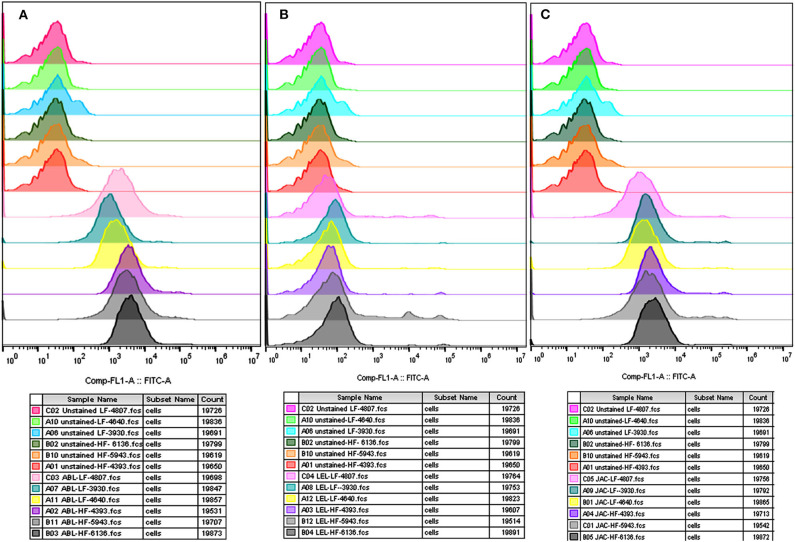
Overlay of the MFI histograms obtained by flow cytometry analysis of spermatozoa from bulls (*n* = 3) of contrasting fertilizing abilities incubated with three FITC-labeled lectins *viz*. ABL **(A)**, LEL **(B)**, and JAC **(C)**. HF, high fertile; LF, low fertile bull spermatozoa.

The flow cytometry was concurrently performed on the spermatozoa from HF and LF buffalo bulls to validate the differential abundance of glycans for ABL, JAC and LEL observed after fluorescence-quantification of lectin binding. The datasets generated from this experiment can be found in the Flow Repository (Rep ID: FR-FCM-Z2GL). The unstained spermatozoa were excluded from the analysis by gating. The flow cytometry for the lectins ABL, JAC, and LEL also produced similar results as were produced by the fluorescence microscopy and the associated fluorescence signal quantification (Figure 3). The average MFI FITC-A values produced by binding of these three lectins were higher in the spermatozoa from the HF bulls in comparison to spermatozoa from the LF bulls (Supplementary Results: Figure 3) albeit non-significantly for JAC and LEL. These results thus validate the differential abundance of the T-disaccharide, galactose of the T antigen, and N-acetyl glucosamine (for ABL, JAC and LEL, respectively) moieties in HF and LF buffalo bull spermatozoa observed during fluorescence signal quantification.

### Differential Lectin Binding Profiles in the Non-capacitated and Capacitated Spermatozoa

**Figure 4:**
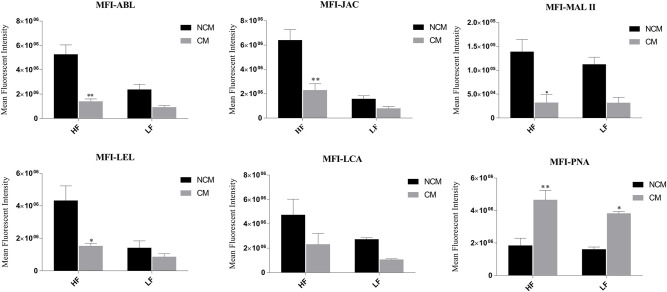
Bar graphs of mean fluorescent intensity (MFI) values obtained after quantification of the fluorescent signal produced during LCC (lectin cytochemistry) experiments using ImageJ. The signal was produced upon binding of six FITC-conjugated lectins *viz*. ABL, JAC, MAL II, LEL, LCA, and PNA on the non-capacitated (NCM) and capacitated (CM) buffalo spermatozoa from bulls (*n* = 3) of contrasting fertilizing abilities. The difference in MFI values was assessed by two-way ANOVA. HF, high fertile; LF, low fertile bull spermatozoa. *P < 0.05 and **P < 0.01.

At the time of capacitation, the sperm surface undergoes extensive remodeling/reorganization especially with respect to the modifications and alterations of the GCs (74). To detect the changes in abundance of sperm glycans after induction of *in vitro* capacitation, the fluorescence signal produced after lectin/s binding were quantified, for the non-capacitated (in NCM) and capacitated (in CM) buffalo bull spermatozoa, in the same manner as already explained. An overall decrease in MFIs, post-capacitation, was observed for five lectins, except PNA in which case the MFI contrarily increased, in both the HF and the LF bulls (Supplementary Results: Figure 4).

In the case of spermatozoa from the HF buffalo bulls, the MFI reduced significantly after capacitation, by 73.38 percentage points upon ABL binding (*P* < 0.01), by 64.20% upon JAC binding (*P* < 0.01), by 64.66% upon LEL binding (*P* < 0.05) and by 76.86% points (*P* < 0.05) upon binding of MALII lectin to spermatozoa surface after the induction of *in vitro* capacitation (Figure 4). Although the MFI reduced by 51.02% points upon LCA binding, the reduction was found to be statistically insignificant. Besides, out of the considered array of six lectins, the MFI of only one lectin i.e., PNA was found to increase significantly in the capacitated spermatozoa from the HF bulls by a whopping 152.46% points (*P* < 0.01) (Figure 4). On the other hand, in the LF bulls, the decrease in the MFI after capacitation was not found to be significant for any lectin. However, for PNA binding there was a significant increase (*P* < 0.05) in the MFI by 137.29 percentage points after capacitation (Figure 4). The results of two-way ANOVA suggested that the interaction of bull fertility status and sperm capacitation status effects was significant for ABL (*p* < 0.05) and JAC (*P* < 0.05) accounting for 11.06 and 13.19% of the total variance, respectively. However, the effects were non-significant for LEL despite a contribution of 13.45% in the total variance. For all the remaining lectins the interaction effects were also found to be non-significant. The capacitation status of the sperm effects accounted for 80.36, 74.76, and 54.23% of the total variance for PNA, MAL-II, and ABL, respectively. The effect of capacitation was also found to be significant for JAC (*P* < 0.05), LEL (*P* < 0.05), and LCA (*P* < 0.05). The effect of fertility status of the bulls was highly significant (*P* < 0.001) for JAC and ABL accounting for 47.64 and 21.65% of the total variance observed in the produced MFI upon lectin binding.

**Figure 5:**
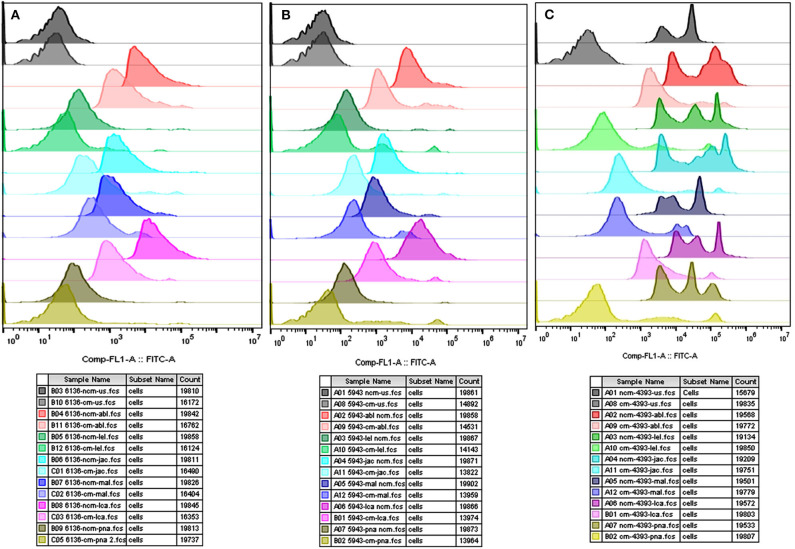
Overlay of the MFI histograms obtained from flow cytometry analysis of non-capacitated (NCM) and capacitated (CM) spermatozoa from three HF bulls *viz*. bull# 6136 **(A)**, bull# 5943 **(B)**, bull# 4393 **(C)** incubated with six FITC-conjugated lectins *viz*. ABL, LEL, JAC, LCA, MAL-II, and PNA. HF, high fertile bull spermatozoa.

The flow cytometry analyses revealed that the average MFI, FITC-A values in the non-capacitated spermatozoa were higher in comparison to the capacitated spermatozoa (Figure 5) for most of the considered lectins in the HF bulls. Due to the large variation in the data, the decrease was deemed statistically non-significant despite a large reduction in MFI values after capacitation. The findings were nevertheless consistent with the results obtained from the LCC experiments on capacitated and non-capacitated buffalo spermatozoa from HF bulls (Supplementary Results: Figure 5). The datasets generated from this experiment can be found in the FlowRepository (Rep ID: FR-FCM-Z2HX).

### Sperm Viability and Lectin Cytochemistry (LCC) After Its Deglycosylation

**Figure 6:**
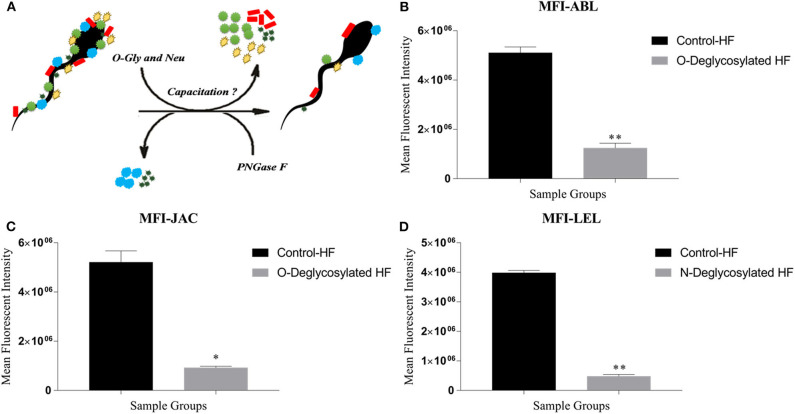
**(A)** The *in situ* removal of O- and N-linked glycan moieties by O-glycosidase along with α2-3,6,8,9 Neuraminidase A (O-Gly and Neu) and PNGase, respectively. **(B–D)** Bar graphs of the mean fluorescent intensity (MFIs) values obtained after quantification of the fluorescent signal produced during LCC (lectin cytochemistry) experiments using ImageJ. The signal was produced upon binding of ABL **(B)**, JAC **(C)**, and LEL **(D)** on the HF and enzyme-treated (Deglycosylated) HF bull spermatozoa. The differences in MFI were assessed by paired two-tailed *t*-test. O-Deglycosylated HF, High fertile bull spermatozoa treated with O-glycosidase along with α2-3,6,8,9 Neuraminidase A, N-Deglycosylated HF, High fertile bull spermatozoa treated with PNGase F. *P < 0.05 and **P < 0.01.

A considerable decrease in MFI, in a time-dependent manner, was observed for the three lectins *viz*. ABL, JAC and LEL after incubating the buffalo spermatozoa with the de-glycosylating enzymes alpha-2,3,4,6,8,9 neuraminidase with O-glycosidase and PNGase F (Figure 6A). This decrease in the MFI was noticeable as early as after 1 h of incubation and continued to decrease with longer incubation times (Supplementary Results: Figure 6). As mentioned earlier, these three lectins were also found to bind differentially on the buffalo spermatozoa from HF and LF bulls. Interestingly, the MFI produced upon LEL binding decreased very rapidly and the visible fluorescence signal disappeared as early as after 1 h of PNGase F incubation.

**Figure 7:**
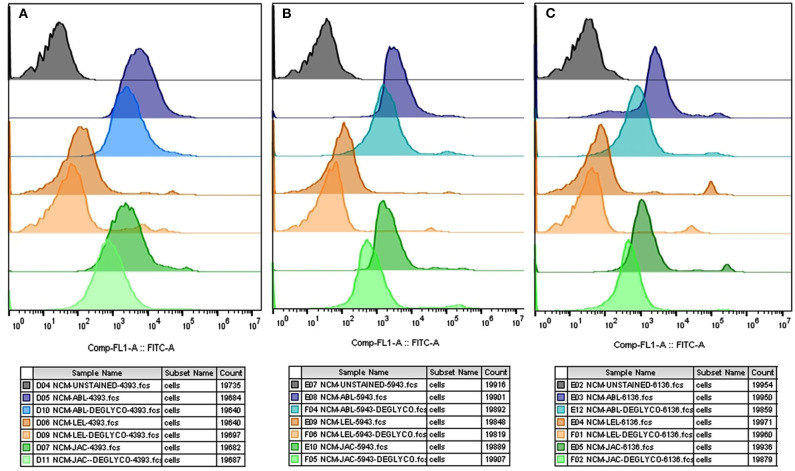
Overlay of the MFI histograms obtained from flow cytometry analysis of intact and deglycosylated bull spermatozoa from three HF bulls *viz*. bull# 4393 **(A)**, bull# 5943 **(B)**, bull# 6136 **(C)** incubated with FITC- conjugated ABL, LEL, and JAC lectins. HF, high fertile bull spermatozoa.

The CFDA/PI staining indicated that sperm viability or the membrane integrity was barely affected after incubating the buffalo spermatozoa with de-glycosylation enzymes for 2 h (Supplementary Results: Figure 7). The live percentage of spermatozoa decreased by only 13.27 percentage points after 2 h of incubation with alpha-2,3,4,6,8,9 neuraminidase and O-glycosidase and 21.22% points after incubation with PNGase F. The decreased percentages of live spermatozoa were not statistically significant from the control group as predicted by one-way ANOVA. Similarly, the percentage of either the moribund or dead spermatozoa didn't vary significantly between the control and the treatment groups (Supplementary Results: Figure 7).

**Figure 8:**
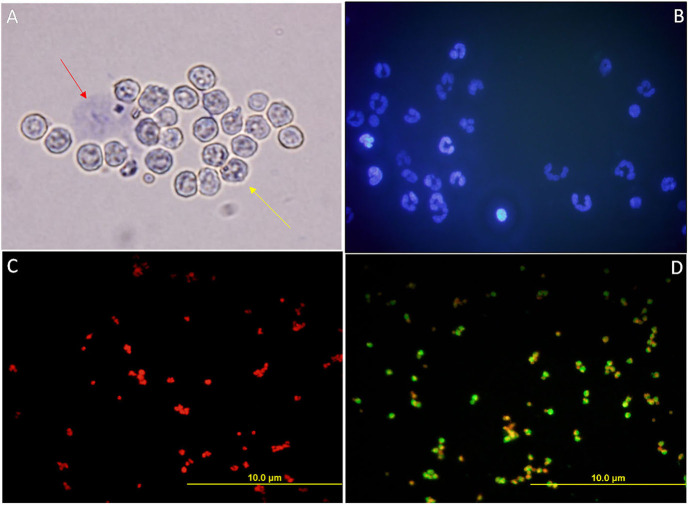
**(A)** Bright-field micrograph of isolated PMN cells from female Murrah buffaloes incubated with Trypan blue stain to assess their viability. Red arrow indicates a dead PMN, the yellow arrow indicates a viable PMN. **(B)** Fluorescent micrograph of the isolated PMNs depicting the hallmark multi-lobed polymorphic nuclei of the PMNs stained with Hoechst 33342. **(C,D)** Fluorescent micrographs of the isolated PMNs subjected to ICC with a monoclonal PMN specific antibody, clone CH138a in conjunction with PI. **(C)** The negative control, PMN without primary antibody. **(D)** Isolated PMN with 1: 200 primary antibody (500 μg/ml).

The MFIs produced upon ABL and JAC binding were significantly lesser by 75.65% points and 82.26% points (*p* < 0.01, *P* < 0.05), respectively (Figures 6B,C and Supplementary Results: Figure 8), in the HF spermatozoa that were de-glycosylated with alpha-2,3,4,6,8,9 neuraminidase and O-glycosidase for 2 h. A large reduction of 88.02% points (*P* < 0.01) in the MFI produced by LEL binding was recorded after treating the spermatozoa from the HF bulls with PNGase F (Figure 6D) for 1 h. Therefore, to ensure maximal removal of glycans moieties, the spermatozoa were incubated with de-glycosylating enzymes for 2 h for JAC and ABL and 1 h for LEL in the subsequent experiments.

**Figure 9:**
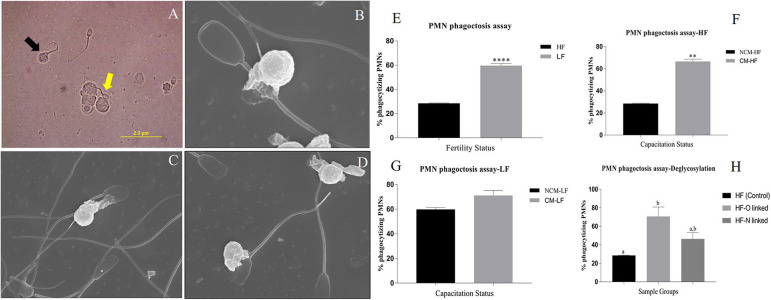
**(A)** A bright-field micrograph (1000X) of the isolated PMNs from female Murrah buffaloes incubated with buffalo bull spermatozoa. Black arrow indicates a phagocytized sperm while the yellow arrow indicates a sperm interacting with PMN. **(B–D)** Electron micrographs (10000X) of isolated PMN from female Murrah buffaloes incubated with buffalo spermatozoa showing phagocytic engulfment of spermatozoa by PMNs. **(E,F)** Bar graphs of percent phagocytizing female PMNs incubated with: **(E)** spermatozoa from buffalo bulls of high and low fertilizing abilities **(F)** spermatozoa from Non-capacitated (NCM) and capacitated (CM) HF bull spermatozoa **(G)** spermatozoa from Non-capacitated (NCM) and capacitated (CM) LF bull spermatozoa **(H)** HF, O-deglycosylated HF (represented by HF-O linked) and N- deglycosylated HF (represented by HF-N linked) bull spermatozoa. HF, high fertile; LF, low fertile bull spermatozoa. **P < 0.01 and ****P < 0.0001.

The flow cytometry analyses revealed that the average MFI FITC-A values for ABL, JAC and LEL in the control HF group were higher in comparison to the treatment (de-glycosylated) group (Figure 7 and Supplementary Results: Figure 9), as was observed in the LCC experiments. The datasets generated from this experiment can be found in the FlowRepository (Rep ID: FR-FCM-Z2HN). The MFIs produced upon lectin binding in the de-glycosylated (either N- or O-linked) HF spermatozoa differed significantly from the control group.

The present data on de-glycosylation revealed that the higher abundance of glycan moieties on the HF sperm could successfully be pruned *in situ* with the de-glycosylation enzymes without significantly affecting the sperm viability. This helped us to develop a de-glycosylated buffalo sperm model for the sperm-neutrophil challenge assay, to elucidate the function of the selected sperm surface glycans in immune-evasion.

### Sperm-Neutrophil (PMN) Challenge Assay

#### Characterization of the Isolated Neutrophil (PMN) Cells From Buffalo Blood

The viability percentage of the isolated PMN cells, as assessed by the trypan blue stain, was more than 90 (Figure 8A). The isolated PMN population had ≥ 95% purity and was found to possess the characteristic multi-lobed nucleus, the hallmark of a PMN cell, as assessed by Hoechst 33342 stain (Figure 8B). Immunocytochemistry, using a lineage-specific marker monoclonal antibody CH138a (anti-CD11) validated the purity of isolated PMNs. (Figures 8C,D).

#### Differential Phagocytosis of High Fertile (HF) and Low Fertile (LF) Bull Spermatozoa by the Neutrophil (PMN) Cells

To know whether a differential SpAGT i.e., the molecular arrangement of glycan moieties in the glycocalyx, is responsible for sperm immune-protection in the FRT, we developed an *in-vitro* phagocytosis model to measure the differential phagocytosis rates of PMN cells incubated with spermatozoa from the bulls of contrasting fertilizing abilities. Since the phagocytosis ensues recognition/adhesion, the spermatozoa interacting with the neutrophils were also included while computing the mean phagocytosis rates (MPRs). The micrographs were acquired using bright field and electron microscopy (Figures 9A–D). Many spermatozoa were found either phagocytized or adhered to the PMN cells through their head or mid-piece or tail. A significantly (*P* < 0.0001) different MPR was observed between the HF and LF sperm. It was found that the mean percentage of phagocytizing PMNs was 28.44 and 59.59% in case of HF and LF bulls, respectively (Figure 9E). More spermatozoa from LF bulls were thus phagocytized by the PMNs *vis-à-vis* the HF bulls. The difference in the MPRs could be ascribed to the differential abundance of glycans observed on the HF and LF sperm-surface. The higher abundance of glycans on the spermatozoa from HF bulls appeared to confer them a superior ability to evade recognition and thus phagocytosis by female phagocytic cells (Neutrophils).

#### Differential Phagocytosis of Non-capacitated and Capacitated Bull Spermatozoa by the Neutrophil (PMN) Cells

To ascertain, whether the changes in the molecular arrangement of glycan moieties (SpAGT) after *in vitro* sperm-capacitation affects its recognition by PMNs, we conducted a sperm-phagocytosis assay using non-capacitated and capacitated spermatozoa. The mean phagocytizing PMNs significantly increased (*P* < 0.01) from 28.44 to 66.49% points after the *in vitro* capacitation of the spermatozoa from HF bulls (Figure 9F). However, the increase in phagocytizing PMNs from 59.69 to 71.00% points after *in vitro* capacitation of the spermatozoa from LF bulls was found to be non-significant (Figure 9G). Overall, a greater number of spermatozoa were found to be phagocytized by the PMNs after *in vitro* capacitation in both HF and LF bulls' spermatozoa.

#### Differential Phagocytosis of Control High Fertile (HF) and the De-Glycosylated High Fertile (HF) Bull Spermatozoa by the Neutrophil (PMN) Cells

The glycocalyx of the mammalian spermatozoa is known to act as an immuno-protectant in the FRT. We hypothesized that the removal of selected glycans increases the proximity of PMNs to the antigenic sites on the sperm-surface, thus increasing the recognition and subsequent phagocytosis. To prove this hypothesis, we again perturbed the normal SpAGT, however, this time by *in situ* de-glycosylation and then performed a PMN–deglycosylated sperm phagocytosis assay. It was found that the de-glycosylated HF spermatozoa were more susceptible to phagocytosis by PMNs *vis-à-vis* the control HF group possessing an intact glycocalyx structure of the inherent SpAGT (Figure 9H). The mean percentage of phagocytizing PMNs in the PNGase-F treated HF bull spermatozoa increased to 46.29% points as compared to 28.4% in the control HF group, albeit non-significantly. However, the MPR increased significantly to 70.63% (*P* < 0.05) in the O-glycosidase & α2-3,6,8,9, Neuraminidase A treated HF spermatozoa. Interestingly, as demonstrated in the LCC experiments there was a significant (*p* < 0.01) reduction of 88.02% points in the MFI by the PNGase-F treated HF spermatozoa, upon LEL binding. Yet, the MPRs were not found to differ significantly from the control HF bull sperm. This indicates that only the O-linked glycans contribute significantly toward the immune-protective abilities of HF spermatozoa.

#### NETosis Assay

**Figure 10:**
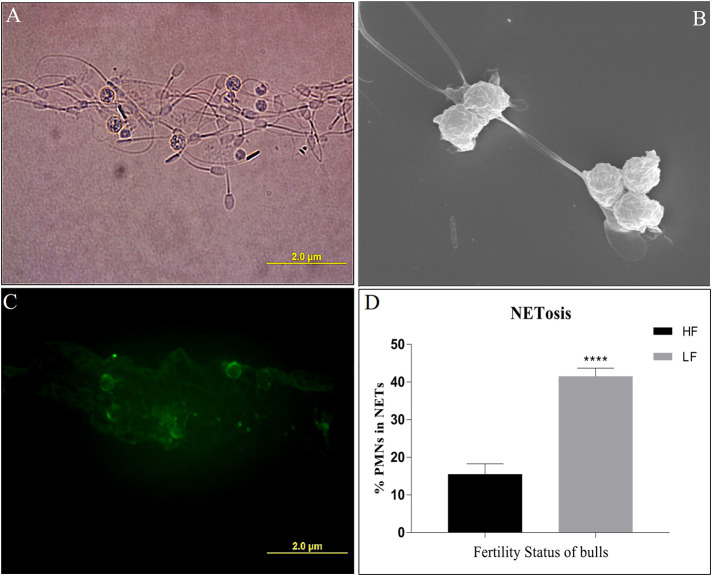
**(A)** Bright-field image of PMNs incubated with spermatozoa depicting NET (Neutrophil Extracellular Trap) formation. **(B)** An electron micrograph of PMNs incubated with spermatozoa depicting the formation of NET. **(C)** A fluorescent micrograph of PMNs involved in the formation of NETs upon exposure to LF bull spermatozoa, obtained by immunocytochemistry with monoclonal anti-MPO antibody. **(D)** Bar graph of per cent PMNs involved in NETosis upon incubation with spermatozoa from bulls of contrasting fertilizing abilities. HF, high fertile; LF, low fertile bull spermatozoa. ****p < 0.0001.

In addition to phagocytosis, the neutrophils follow another effector mechanism to combat and kill the foreign pathogens by trapping them in their nucleic acid and producing mesh-like structures known as Neutrophil Extracellular Traps (NETs). To assess the NET formation by the PMN cells, we challenged the HF and LF buffalo sperm with PMNs using the same *in-vitro* challenge model, however, extending the incubation time (180 min). The immunofluorescence (anti-MPO), electron and micrographs revealed that the buffalo neutrophils formed cluster-like structures of varying sizes, which consisted of PMNs and buffalo spermatozoa ensnared in NETs (Figures 10A–C). We detected the phenomenon of NET formation (NETosis) in both the groups of spermatozoa, albeit at differential rates. Our data showed a significant (*p* < 0.0001) difference in the percentage of PMN cells forming NETs when incubated with spermatozoa from both the fertility groups. Notably, the percentage of PMN cells forming NETs when incubated with spermatozoa from LF bulls was 41.47 *vis-à-vis* 15.46%, when incubated with spermatozoa from HF bulls (Figure 10D).

### RT-qPCR for NETosis

**Figure 11:**
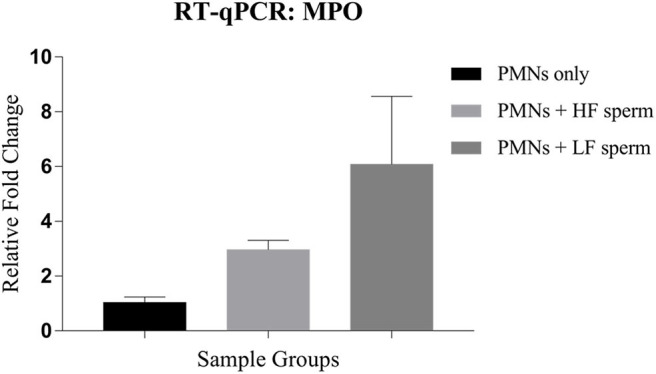
Relative expression profile of MPO in the PMNs only (control group) *vis-à-vis* the groups where the PMNs were challenged with spermatozoa from the bulls of contrasting fertilizing abilities. HF, high fertile; LF, low fertile bull spermatozoa.

The neutrophil elastase (NE) and myeloperoxidase (MPO) are the PMN granular enzymes essential for neutrophil extracellular trap (NET) formation. We assessed the transcript abundance of only the MPO during NET formation because NE is stored in the precursor stages of their lineage and isn't known to be transcribed *de novo* after maturation is attained. Our results indicated that the expression of MPO was 6-fold higher when the PMNs were incubated with the spermatozoa from LF bulls compared to the control group (PMNs without spermatozoa). On the other hand, the increase in expression was only 3-fold with the spermatozoa from the HF bulls (Figure 11). The increase in transcript abundance was found to be non-significant in both groups. Overall, our findings demonstrated that the transcript abundance of MPO correlated with the observed NET formation after incubating the PMNs with spermatozoa from bulls of varying CRs.

## Discussion

The present work was undertaken to find out whether the spermatozoa from the bulls of contrasting fertility differ in their glycans abundance or glycocalyx composition and hence in their immune-evasion properties. We propose that the glycans abundance affects the fixed, 3-D spatial arrangements of the monosaccharide units of the glycocalyx and therefore its rendered configuration, which we address as the Sperm Associated Glycan Topography (SpAGT). We aimed to demonstrate a differential abundance of glycans on HF and LF bull spermatozoa and to investigate the effect of this differential or a perturbed SpAGT on neutrophil recognition, phagocytosis and NETosis. The difference in the manifested SpAGT is expected to influence the downstream information transfer by the sperm glycocalyx to the PMN cells. Therefore, we hypothesized that a differential or perturbed SpAGT would affect intercellular communication between buffalo spermatozoa and the female PMNs and thus mediate different modalities of the PMNs. It was demonstrated that the HF and LF spermatozoa possess a differential abundance of N-and O-linked glycans, indicating a differential SpAGT manifestation. This differential abundance (SpAGT) appeared to determine the rates of phagocytosis and NETosis upon *in vitro* stimulation of PMNs by the buffalo spermatozoa. Besides, any perturbations in the glycocalyx structure (SpAGT) e.g., by removal of O-linked glycans (*in situ* de-glycosylation) or by inducing *in vitro* capacitation, appeared to elevate the rates of binding (recognition), phagocytosis and NETosis effector mechanisms displayed by the buffalo neutrophil cells. We propose that the differential or perturbed SpAGT varying in O-linked glycan abundance reflects the molecular and structural modifications in the sperm glycocalyx, which modulates the normal functions of the communication in the FRT. This study would help to better understand the role of the glycans in facilitating sperm survival in the face of innate immunity.

The glycoconjugates (GCs) of the glycocalyx are crucial components of several molecular mechanisms which underlie the regulation of interactions between the spermatozoa and the various cells and impediments encountered by the spermatozoa during its transit in the immunologic milieu, across the FRT (5, 19, 77, 78). A spatial variation in the abundance of a variety of glycan moieties of the GCs was observed on the buffalo sperm surface in this study. The differential distribution of the selected sugars was recognized by the regions of the restricted fluorescence (micro-domains) produced upon binding of six FITC-labeled lectins on the buffalo sperm-surface (Figure 1). The lectins are not only known for their glycan recognition abilities but also are used for *in situ* identification of linkage and branching pattern, determination of their spatial distribution and localization patterns and their quantification (2, 26, 79–82). The various glycans moieties on the plasma membranes are capable of forming clustered saccharide patches such as the sialylated clusters and glycosynapses which contain O-linked glycans on the membrane surface (83, 84). The observed restriction of fluorescence signal to the mid-piece upon MALII binding, to the acrosomal and post-acrosomal regions upon LCA and LEL binding and along the periphery of buffalo spermatozoa upon JAC and ABL binding indicated that these glycan micro-clusters or patches may mediate specific spatial interactions in adhesion, recognition, signal transduction and motility of the buffalo spermatozoa, as reported for other cells (85, 86). The spatial distribution pattern of glycans has also been demonstrated to affect the stability and behavior of cell membranes (86, 87). The LEL specific GCs have recently been reported to exist as clusters in a confined region (88). The region-specific micro-heterogeneity in the abundance of T disaccharide i.e., galactosyl (β-1,3) N-acetylgalactosamine, galactose of the T antigen, and N-acetyl glucosamine (cognate glycans for ABL, JAC and LEL, respectively) observed in the lectin cytochemistry experiments reflect distinctive and spatially distributed, putatively functional micro-domains on the buffalo sperm. The pattern of fluorescence produced upon JAC binding needs a special mention because of the ability of Jacalin to bind with both mono- as well as di-sialylated forms of galactosyl (β-1, 3) N-acetylgalactosamine. The observed differential binding patterns in HF and LF semen samples could be due to two sub-populations of spermatozoa differing in the abundance of sialylation in galactosyl (β-1, 3) N-acetylgalactosamine (1, 4). It appears to be the case because these patterns were differentially present in the spermatozoa from HF and LF bulls, although the differences were non-significant (Supplementary Results: Figure 10). Multiple patterns of lectin staining have earlier been reported in the spermatozoa of many species including bovine (74), bats (13), human and mouse (56, 75, 89, 90) providing evidence for the existence of sub-populations in the ejaculated semen. Moreover, the pattern of lectin binding observed in this study differs from what has been observed in cattle and humans (3, 74, 75) highlighting species-specific glycan distribution on sperm-surface. Accruing evidence has indicated that the plasma membrane is laterally compartmentalized suggestive of a nanoscale organization of various membrane microdomains. Largely, the spatial restriction and the localization patterns of the glycans observed in buffalo spermatozoa indicated the existence of various glycan micro-domains having potential region-specific physiological or functional specialization (4, 5).

We also found that the cognate-glycans for the lectins ABL, JAC and LEL *viz*. galactosyl(β-1,3)N-acetylgalactosamine of the T antigen, galactose of galactosyl(β-1,3)N-acetylgalactosamine and [GlcNAc]1-3, N-acetylglucosamine were differentially abundant in the spermatozoa of buffalo bulls with contrasting CRs. Our results revealed the existence of a higher abundance of not only the O-linked but also the N-linked glycans on buffalo spermatozoa from HF bulls in comparison to the LF bulls (Figure 2). Numerous O-linked glycans are added on the sperm surface primarily in the epididymal lumen, which has aptly been given the epithet “the extracellular Golgi,” while many N-linked glycans are known to be lost from the sperm-surface during this time (91, 92). Any incongruity in the SpAGT of the sperm glycocalyx is likely to influence its molecular function/s because a different conformational arrangement of glycans around the sperm-surface is expected to affect the downstream information flow of a cell (61). For example, in primates, the mutant DEFB-126 spermatozoa with an impaired glycocalyx were found to bind a lesser number of ABL and JAC lectin molecules compared to the sperm with wild type DEFB-126 glycoprotein. A lesser quantity of the O-linked glycans was found to compromise the ability of the DEFB-126 mutant sperm to penetrate the cervical mucus (3, 14, 36). Similarly, a differential PNA binding has been associated with the fertility status of the HF and LF bulls, indicating the differential abundance of asialylated galactosyl (ß-1, 3) N-acetylgalactosamine in their spermatozoa (79, 93, 94). Likewise, a decrease in the sialic acid content from the cattle bulls was found to decrease the motility and the CMP ability, nevertheless, raised the incidence of polyspermic penetration of the eggs (19, 77). A differential or probably anomalous spatial arrangement of O-linked and N-linked glycans, for example, those associated with the LF-SpAGT, is expected to render a distinct conformation on the spermatozoa of HF bulls. This difference is anticipated to affect the molecular functions of SpAGT in the FRT, considering the vast amount of information like the identity of “self,” age and gene expression history stored in the glycocalyx (61, 64).

The dramatic biochemical and extensive modifications including the re-organization and alteration of the GCs that occurs during capacitation prepare the sperm for successful fertilization (7, 36, 74, 95). In the present study, a significant reduction in the MFI upon JAC and ABL, MAL II and LEL binding was observed in the *in vitro* capacitated spermatozoa *vis-à-vis* the non-capacitated spermatozoa of the HF buffalo bulls. This decrease indicated a major loss of the O-linked and N-linked glycans on the buffalo sperm-surface, after *in vitro* capacitation. It is well known that during sperm capacitation most of the surface glycoproteins are extensively modified wherein most O-linked and a few N-linked glycans are removed (4, 96). The observed loss of oligosaccharides from the sperm-surface of HF buffalo bulls in the post capacitated spermatozoa (Figures 5, 6) could also indicate loss of non-covalent interactions between the sperm and the molecules acquired during the epididymal transit (93). It, however, remains undetermined whether the observed decrease in the MFI after *in vitro* capacitation was the result of the re-arrangement or displacement or loss or a conformational change in glycans associated with the GCs on the sperm surface. Nonetheless, no change in the localization pattern was observed after capacitation. Numerous reports are available on the changes in lectin binding after *in vitro* capacitation spermatozoa in various species like cattle (75), boar (97) and mice (90). To validate that the loss of MFI associated with capacitation was actually due to the loss of glycan moieties, the HF spermatozoa were treated with O-glycosidase along with α2-3,6,8,9 Neuraminidase A and PNGase F. The MFI produced upon the ABL, JAC, and LEL binding decreased significantly, without any changes in the localization pattern in a time-dependent manner after incubation with de-glycosylation enzymes. These results underpinned the proposition of progressive removal/re-organization of their cognate receptor ligands of the lectins (glycans) from the sperm plasma membrane after capacitation. The changes in the structures of the GCs i.e., the changes in the molecular arrangements of the glycocalyx (SpAGT) associated with perturbations such as capacitation (11, 74), cryopreservation (8, 34), flow cytometry (35, 74), or removal of glycans by using enzymes (8, 53) have successfully been monitored and well documented using lectins as reporters. Overall, our data reveal that both the O-linked and N-linked glycans were either removed or extensively re-organized during the *in vitro* induced capacitation of the buffalo spermatozoa, thus possibly affecting the overall topography of its glycocalyx.

To determine the role of glycans in immune evasion we incubated the HF and LF bull spermatozoa with the PMNs isolated from female buffaloes presumably simulating *in vivo* conditions. The insemination or intromission of any liquid in the uterus has been found to trigger multiple immune mechanisms that assist in sperm selection and fertilization of the egg by the spermatozoa (98). It also initiates *en-masse* recruitment of the leukocytes in the uterine lumen through the sub-epithelial stroma, known as the leukocyte reaction. The recruited cells majorly include the foot soldiers of the innate immune system, the neutrophils or PMNs. This influx has been shown to bring demise to the majority of the inseminated allogenic spermatozoa population in the FRT (53, 56, 99–103). The observed proximity or interactions between the spermatozoa from HF or LF bulls and the isolated PMNs suggest the involvement of cognate adhesion molecules e.g., the PRRs on the PMNs for binding the glycans on the buffalo spermatozoa (Figure 9). Interestingly, the HF spermatozoa were found to be better at evading the neutrophil recognition and the ensuing phagocytosis *vis-à-vis* the LF spermatozoa. The acuity of buffalo neutrophil PRRs in recognition of the uniquely manifested SpAGT of HF and LF bull spermatozoa is likely to be responsible for their differential spermiophagy rates. The mammalian spermatozoa are known to acquire various immune-suppression molecules during their journey in the epididymis some of which are required for immune-evasion in the FRT (25). We propose that GCs are a crucial component implicated in modulating the immune responses mounted in the FRT against the allogenic spermatozoa. The incidences of observed spermiophagy were nearly twice for the spermatozoa of the LF bulls compared with the HF bulls. The observed difference in the sperm phagocytosis could be ascribed to the lower abundance of glycans in LF-SpAGT than that of the HF- SpAGT, which appears to transduce a different recognition signal presumably to the bound PRRs of the interacting neutrophils. A higher abundance of SLe^x^ and other tri- and tetra-antennary glycans have been reported in cancer patients, resulting in lack of immune-detection of tumor cells (46). Thus, in accordance with the “Glyco-evasion hypothesis” (46), a higher abundance of glycans on HF bull sperm modulates the host (FRT) immune response resulting in higher incidences of immune-evasion in such spermatozoa. There are many reports of spermiophagy by neutrophils in diverse species like mice (5), boar (58), bovine (22, 55, 104), human (11) and equine (30) where it had been demonstrated that a spermatozoon can provide the threshold stimulus required to trigger the PMN activation similar to lectinophagocytosis as observed in the case of buffalo in this study (29, 70). Given the fact that no explicit opsonins were added to the *in vitro* immune challenge assay used in this study, we propose the lectin-carbohydrate dependent interaction could be the possible mechanism behind buffalo sperm-neutrophil interaction/s. The proposed mechanism appears valid because the LF-SpAGT exposure elevated the observed incidences of spermiophagy *vis-à-vis* the HF-SpAGT. Every glycan moiety in the SpAGT of the glycocalyx thus seemingly carries potential information about its identity. It appears that due to higher glycan abundance, the HF-SpAGT indicatively could function better as SAMPs in evading the adhesion and spermiophagy by the neutrophils more effectively than the LF-SpAGT (105).

**Figure 12:**
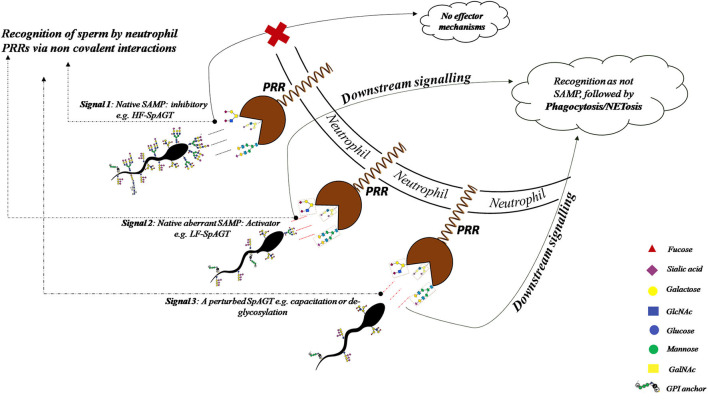
The recognition of HF-SpAGT presumably by the neutrophil PRRs appears to be inhibitory for effector mechanisms like spermiophagy and NETosis, while the LF-SpAGT or a perturbed inherent SpAGT, activates the neutrophils thus bringing demise to such buffalo spermatozoa.

**Figure 13:**
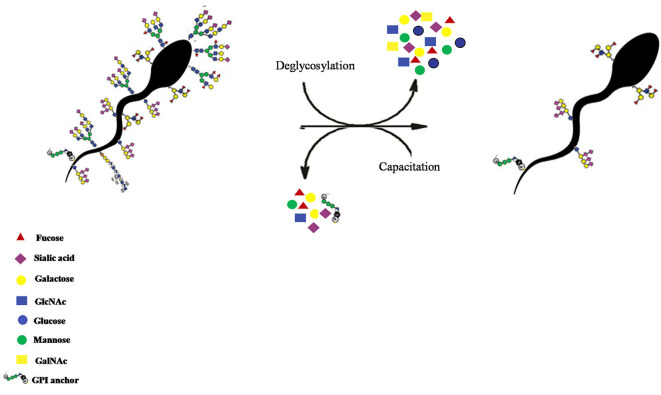
The removal of the buffalo sperm-surface GCs after *in situ* deglycosylation with deglycosylation enzymes like α2-3,6,8,9 Neuraminidase A, O-glycosidase and PNGase F treatment or by *in vitro* capacitation of the buffalo spermatozoa.

The perturbations in the SpAGT occurring after the *in vitro* capacitation elevated the observed incidences of neutrophil phagocytosis presumably by either increasing the number of available binding sites (glycans on sperm) or by presenting different recognition signals, to the bound PRRs and/or adhesion molecules present on the PMNs (Figures 12, 13). While we report an increase in the number of PMNs performing spermiophagy in the *in vitro* capacitated spermatozoa of the HF and LF bulls, Tecle et al. recently reported that sperm capacitation did not affect *in vitro* neutrophil activation in humans (11). On the other hand, a decrease in spermiophagy after *in vitro* capacitation of porcine spermatozoa had previously been reported (58, 100). These species-specific differences could be imputed to the high variability in the glycan distribution and differential glycocalyx structure amongst various species (4, 23, 68, 99). Thus, the unique SpAGT of the buffalo spermatozoa glycocalyx appeared to confer or regulate a different response modality of the neutrophil activity after *in vitro* capacitation. It is possible that in the non-capacitated spermatozoa, the antigenic sites are concealed under the intact dense sugar arrays (glycocalyx), which has been compared to the Klingon cloak making them imperceptible to the immune system in the FRT till they reach the oviduct (14). It has been proposed that either the post-capacitated spermatozoa are prone to the phagocytosis or act as altruists engaging the leukocytes in an attempt to assist other sperm to march further in the oviduct (60, 106, 107). It is noteworthy that the leukocyte reaction has been implicated in the reduction of male fertility rates since it interferes with the normal sperm transport in the FRT. It is especially relevant in the case of AI where the most of the seminal plasma, which regulates the PMN activity in FRT, is lost during semen processing, as mentioned earlier (23, 30, 55).

The major part of the female genital tract appears to have devised many selection criteria to choose only those sperm which are alive, morphologically normal with an intact DNA so that a particular spermatozoon can ultimately fertilize the egg (60, 66). Although the glycocalyx structure has been proposed to be the molecular frontier implicated in “cryptic female choice,” no information exists correlating the glycocalyx variation with the fertilizing ability of the spermatozoa, until recently (65, 108, 109). As observed in the present study, the perturbation in SpAGT by the removal of O-linked glycans from buffalo sperm glycocalyx elevated the observed MPRs significantly. Alterations in the cell-surface glycans are well known to lead to defects in cellular recognition and motility (88, 110, 111). It has already been demonstrated that the glycocalyx of the sperm acts as an immune protecting coat that helps the spermatozoa in the FRT to evade the immune responses mounted against it (4, 14, 59, 112). Yet, the molecular and mechanistic details of the sperm-PMN interactions remain largely obscure. The intact HF or LF spermatozoa and the spermatozoa with introduced perturbations in the molecular arrangement of the GCs (by capacitation or enzymatic deglycosylation) appeared to manifest a unique topography out of their available glycans moieties (Figures 12, 13). This topography, as mentioned previously, appeared to be specified by the region-specific abundance of individual glycans and their spatial arrangement rendered in the glycocalyx present on the buffalo sperm surface. Such perturbations in the SpAGT are expected to affect its molecular function of information transduction in the FRT as observed by the increased neutrophil recognition and spermiophagy of deglycosylated spermatozoa, reported in this study (Figure 12). Interestingly, the O-linked glycans associated with DEFB-126 glycoprotein on the macaque spermatozoa have been reported to protect them from immune-recognition inside the FRT (14, 21, 112). Likewise, the removal of sialic acid from the sperm-surface by sialidase has been reported to increase the phagocytosis by neutrophils (5) and by macrophages (59). This removal was also found to affect the sperm motility and its cervical mucus and egg penetration abilities, eventually affecting the fertilization capability of the sperm (19). The oligo-mannosidic chains, nonetheless, have been proposed to act as a recognition signal for eliminating the incompetent sperm during their transit in the FRT (26, 113). Interestingly, the removal of the N-linked glycan moiety, [GlcNAc]1-3, N-acetylglucosamine was not found to affect the MPR significantly. This suggests that the N-linked glycans of the buffalo spermatozoa might not contribute much in evading from immune-recognition by the neutrophils. We therefore, propose that either a lesser abundance of specific glycocalyx constituents (O-linked glycans) or the selected removal or re-arrangement of its constituents affects the inherent SpAGT of the buffalo spermatozoa. This results in increased proximity of PMNs to the existing antigenic sites on the buffalo spermatozoa surface. This aberrant/perturbed SpAGT seemingly couldn't effectively function as SAMPs, thereby bringing about neutrophil phagocytosis of the capacitated, de-glycosylated and LF bulls spermatozoa (Figure 12).

The PMNs/neutrophils are also armed with other antimicrobial effector strategies apart from the phagocytosis like degranulation and NETosis (114–116). The immunofluorescence and the electron micrographs generated from our work demonstrated that the neutrophils formed differentially sized clusters consisting of the neutrophils and the enmeshed spermatozoa as has been observed for human sperm (117). The incidences of such clusters were nearly twice more for the spermatozoa from LF bulls in comparison to the spermatozoa from the HF bulls. Our findings indicated that the spermatozoa from the HF bulls are better at evading enmeshment in these extracellular traps *vis-à-vis the* LF bulls. A differential SpAGT is likely to be the key factor in elevated entrapment and subsequent demise of spermatozoa from LF bulls via NET formation. The phenomenon of NETosis is implicated in the regulation of bull fertility (23). Many PTMs including glycosylation have been reported to differentially modulate the NET formation, indicating the role of SpAGT in evading NETosis and the subsequent spermiophagy by the neutrophils (99, 101, 118–120). This indicates that the LF-SpAGT is perceived as a distinct arbiter of the neutrophil response by the PRRs of the neutrophils *vis-à-vis* the HF-SpAGT. This study implicates O-linked glycans in evading the innate immune responses mounted by PMNs against the allogenic spermatozoa and also provides a theoretical basis for explaining idiopathic male infertility with unknown reasons. This is because the common tests for the sperm fertilizing ability such as motility, viability and morphology assessment don't always identify the sub-fertile ejaculate. Furthermore, a high inter-lab and inter-technician variability have been reported using “strict morphology” as the predictor of the fertility status (121). Therefore, novel means to assess the fertilizing ability should be incorporated in the existing SOPs at semen stations so that freezing of sub-fertile spermatozoa can be avoided. In these lines, the present study recommends the abundance of the O-linked glycans on the buffalo spermatozoa as one of the novel criteria for that could be used for sperm fertilizing ability assessment to achieve higher reproductive efficiency in livestock production.

## Conclusion

The study concludes that a differential abundance of galactosyl(β-1,3)N-acetylgalactosamine and [GlcNAc]1-3, N-acetylglucosamine exists between the spermatozoa from HF and LF bulls which brings about the differential response by the female neutrophils. A lesser abundance of O-linked glycans appears to act as the recognition signal regulating the sperm survival by modulating the sperm adhesion followed by spermiophagy and/or NETosis by the neutrophils. Our results confirm that the *in vitro* exposure of live spermatozoa to the PMNs provides the threshold stimulus that leads to activation of the two effector mechanisms exhibited by the PMN cells viz. phagocytosis and NETosis. The concept of SpAGT can provide new insights into the molecular rearrangement (e.g., branching, linkage and identity) of the glycans on the sperm surface and their functional significance in PMN effector mechanisms. The results presented in this study appear to corroborate the suggestion that the neutrophils actively take part sperm selection removing non-competent, incompetent spermatozoa and thus, spermiophagy appears to be a selective rather than a random process. Although we have just begun to understand the information stored in the sperm glycome, formulating an extensive molecular cartograph elucidating the entire SpAGT is mandatory to harvest and decode the vast amount of information stored in the sperm glycome. To the best of our knowledge, this is the first attempt to associate O-linked glycans with the fertility status of the spermatozoa. Nonetheless, further studies with a higher number of animals are required to clarify the correlation between the sperm glycocalyx structure and its encoded information about survival in the FRT upon neutrophil encounter.

## Data Availability Statement

The datasets generated from this study can be found in the FlowRepository (Rep ID: FR-FCM-Z2GL, FR-FCM-Z2HX, and FR-FCM-Z2HN).

## Ethics Statement

The animal study was reviewed and approved by Institutional Animal Ethics Committee (IAEC), National Dairy Research Institute.

## Author Contributions

The study was conceptualized and designed by TD, VB, KD, and RK. Sample collection & expression analysis was done by and KD and VB. AK and TD performed statistical analyses. SN assisted in LCC. The manuscript was written by VB. Figures were designed by AK and KD. All authors contributed to the article and approved the submitted version.

## Conflict of Interest

The authors declare that the research was conducted in the absence of any commercial or financial relationships that could be construed as a potential conflict of interest.
