# Supplementary material for: A Higher Abundance of O-Linked Glycans Confers a Selective Advantage to High Fertile Buffalo Spermatozoa for Immune-Evasion From Neutrophils
Source: Front Immunol. 2020 Aug 28;11:1928. doi: 10.3389/fimmu.2020.01928 (PMC7483552; doi:10.3389/fimmu.2020.01928)
Supplement: Supplementary file 1 [file Data_Sheet_1.docx]

Supplementary Material

**Supplementary Methods-Table 1: List of selected bulls and their overall conception rates (CRs)**

| **Fertility Groups** | **Buffalo bull ID number** | **Total number of AIs** | **Conceived Buffaloes** | **Conception Rate (%)** | **Number of available straws** |
| --- | --- | --- | --- | --- | --- |
| Low Fertile (LF) | 3930 | 123 | 26 | 21.14 | 1532 |
| Low Fertile (LF) | 4807 | 73 | 13 | 17.81 | 1992 |
| Low Fertile(LF) | 4640 | 85 | 23 | 27.06 | 783 |
| High Fertile (HF) | 6136 | 99 | 45 | 45.45 | 6698 |
| High Fertile (HF) | 5943 | 78 | 37 | 47.44 | 3740 |
| High Fertile (HF) | 4393 | 121 | 60 | 49.59 | 2573 |

**Supplementary Methods-Table 2: Composition of the capacitating sp-TALP media (CM) and non-capacitating TALP media (NCM)-2x stock**

| **Component** | **CM** | **NCM** |
| --- | --- | --- |
| **NaCl** | 100 mM | 100 mM |
| **HEPES** | 10 mM | 10 mM |
| **KCl** | 3.1 mM | 3.1 mM |
| **EDTA** | 0.4 mM | 0.4 mM |
| **MgCl_2_.6H_2_O** | 0.4 mM | 0.4 mM |
| **NaH_2_PO_4_.2H_2_O** | 0.3 mM | 0.3 mM |
| **BSA** | 6mg/ml | **-** |
| **Cacl_2_.2H_2_O** | 2mM | **-** |
| **NaHCO_3_** | 10mM | **-** |

**Supplementary Methods-Table 3: List of selected FITC-conjugated lectins and their standardization concentration range. Each lectin stock was diluted in PBS at various working concentrations (10 to 125 µg/ml) to find the desirable concentration that would produce the highest signal with lowest background noise, near the saturation point of fluorescent signals.**

| **Lectins** | **Concentrations range (µg/ml)** |
| --- | --- |
| - LEL | 10 – 100µg/ml (10 , 15 , 25 , 50 , 75 and 100 µg/ml) |
| - ABL | 15 -125 µg/ml (15 , 25 , 50 , 75 , 100 and 125 µg/ml) |
| - JAC | 10 - 125 µg/ml(15 , 25 , 50 , 100 and 125 µg/ml) |
| - LCA | 10 - 100 µg/ml (5 , 15 , 20 , 50 and 100 µg/ml) |
| - MAL II | 10 -100 µg/ml (10 , 15 , 20 , 50 , 75 and 100 µg/ml) |
| - PNA | 10 - 100 µg/ml (10 , 15 , 25 , 50 , 75 and 100 µg/ml) |

**S.1 Standardization of enzyme concentrations incubation time and membrane integrity test**

Optimum concentrations of different enzymes (as per manufacturer’s instructions) were used and incubated with buffalo spermatozoa for different time intervals i.e. 0, 1, 2,3 and 4 hours in a 5% CO_2_ incubator (37^o^C) to finalize the concentration at which a significant decrease in fluorescence intensity because of glycans removal was observed without hampering the sperm cell membrane integrity. Thereafter, lectin cytochemistry was done using de-glycosylated spermatozoa samples, as explained in the text. After the deglycanase treatment for selected time points, the buffalo spermatozoa were subject to CFDA (carboxyfluoresceindiacetate)-PI dual staining for assessment of membrane integrity. Briefly, 10 x 10^6^ post-swim-up spermatozoa in working-NCM (40µl) were incubated with 15µl of CFDA for 14 min at 37°C in dark in1.5 ml microcentrifuge tubes (MCTs) and 1ul PI was added. The samples were then washed, smeared on glass slides onto which one drop of mounting medium, Dabco® 33-LVwas placed before putting cover-slip on the air-dried slide and were observed at 1000X magnification under Olympus BX-51 fluorescence microscope under Blue-filter for CFDA and Green-filter for PI.

**S.2 Preparation of pure PMN cells from blood**

Briefly, 5 ml of Polymorphprep was taken in a 15 ml falcon tube and 5 ml of undiluted blood was slowly layered over the Polymorphprep solution and mixture was centrifuged at 500g for 30 min at room temperature. The plasma and mononuclear cells (the upper band of cells) were removed and PMNs in the lower band of cells were carefully harvested. The neutrophil solution was diluted with half-concentration of Hepes-buffered saline that consisted of 0.85% (w/v) NaCl and10 mM Hepes-NaOH, pH 7.4. The suspension was mixed gently followed by centrifugation at 400 x g for 10 min at RT. A red pellet appeared which was suspended in the Hepes-buffered saline. This pellet was re-suspended in 3 ml of Ammonium chloride lysis buffer (0.83% (w/v) NH_4_Cl, 10 mM Hepes-NaOH), pH 7.4 and kept for 7 min at 37°C to lyse the erythrocytes. Subsequently, the PMNs were pelleted by centrifugation for 5 min at 2000 rpm and the lysis procedure was repeated once again in ammonium chloride lysis buffer. Each neutrophil pellet was washed in suitable media (TCM-199, TALP) and re-suspended in it according to the subsequent experiment requirements.

**S.2.1 Morphological characterization of isolated PMNs**

Nuclear stain Hoechst 33342 was used to observe the multi-lobed nucleus of the neutrophil cells. Briefly, 100µl of the PMN cells was mixed with 20µl of Hoechst 33342 stain to stain the nucleus and kept in dark for 15 min. Cells were subsequently centrifuged for 2 min at 300 x g and the staining solution was aspirated completely. The cells were then washed twice with PBS. The neutrophils were observed at 1000X magnification under BX-51 Olympus fluorescence microscope on a glass slide.

**S.2.2 Antigenic characterization of isolated PMNs**

Cultured neutrophils (80 x 10^6^cells/ml) were re-suspended in DMEM media and were seeded into a 6-well microtiter plate containing a poly-L-lysine coated cover-slip. After 30 min.the media was removed and the cells were washed twice in PBS and fixed in 4% paraformaldehyde (PFA) for 20 min at room temperature. The cells were then washed with PBS thrice, and the plate surface was blocked with blocking buffer (1% BSA in PBST) for 1 hour at room temperature (RT). Cells were incubated with primary monoclonal antibody CH-138A (10µg/ml) against neutrophil specific marker CD11c overnight at 4°C. The cells were washed with PBST thrice and were subsequently incubated with FITC conjugated anti-mouse IgG secondary antibody (1µg/ml) in dark for 1 hour at RT followed by final washings with PBST (3x). Additionally, all the cells were treated with PI for 1 min at RT to stain the nuclei after washing of secondary antibody with 1X PBST three times. After final washing, the coverslip was mounted onto a dried glass slide onto which one drop of mounting medium, Dabco® 33-LVwas placed. The cells were then observed under BX-51 Olympus fluorescence microscope.

**S.3 RNA extraction from sperm-challenged PMNs and control groups**

The PMN cells exhibiting NETosis were collected and re-suspended in 100µl of PBS in 1.5 ml MCTs. The cells were mixed with 900 µl of Trizol reagent (Invitrogen) as per manufacturer’s instructions. The RNA pellet was dried in the air followed by dissolved in 30 µl of DEPC treated water and placed in a heating block at 55ºC for 5 min. Extracted RNA was quantified using a NanoDrop ND-1000 UV–Vis Spectrophotometer (NanoDrop Technologies Inc., Wilmington, DE, USA). The quality and integrity of extracted RNA were assessed by running 200ng of RNA (heated at 65°C for 1 minute) in non-denaturing TAE buffered 1.2% agarose.

**S.3.1 Preparation of cDNA**

RevertAid, H Minus First Strand cDNA Synthesis Kit (Thermo Scientific, USA) was used to convert 2µg of RNA into cDNA as per manufacturer’s instructions. A reverse transcriptase negative (RTN) control was prepared for detection of residual genomic DNA contamination in the RNA sample.

**S.3.2 Primer design and expression analysis of NETosis responsible enzymes**

The primer designing tool of NCBI, the Primer-BLAST was used to design primers for the MPO (Myeloperoxidase) and two reference genes viz., eEF-2 (eukaryotic elongation factor 2) and RPS-18 (Ribosomal protein S18). Intron-spanning primers were designed and the self-annealing sites, mismatches and secondary structures in the primers were checked using Oligonucleotide properties calculator. The specificity of primers was again checked using BLAST alignment tool and *in silico*, PCR was run for each set of primers before commercial synthesis (Sigma-Aldrich, USA).
