# Supplementary material for: A Higher Abundance of O-Linked Glycans Confers a Selective Advantage to High Fertile Buffalo Spermatozoa for Immune-Evasion From Neutrophils
Source: Front Immunol. 2020 Aug 28;11:1928. doi: 10.3389/fimmu.2020.01928 (PMC7483552; doi:10.3389/fimmu.2020.01928)
Supplement: Supplementary file 2 [file Presentation_1.PPTX]

## Slide 1
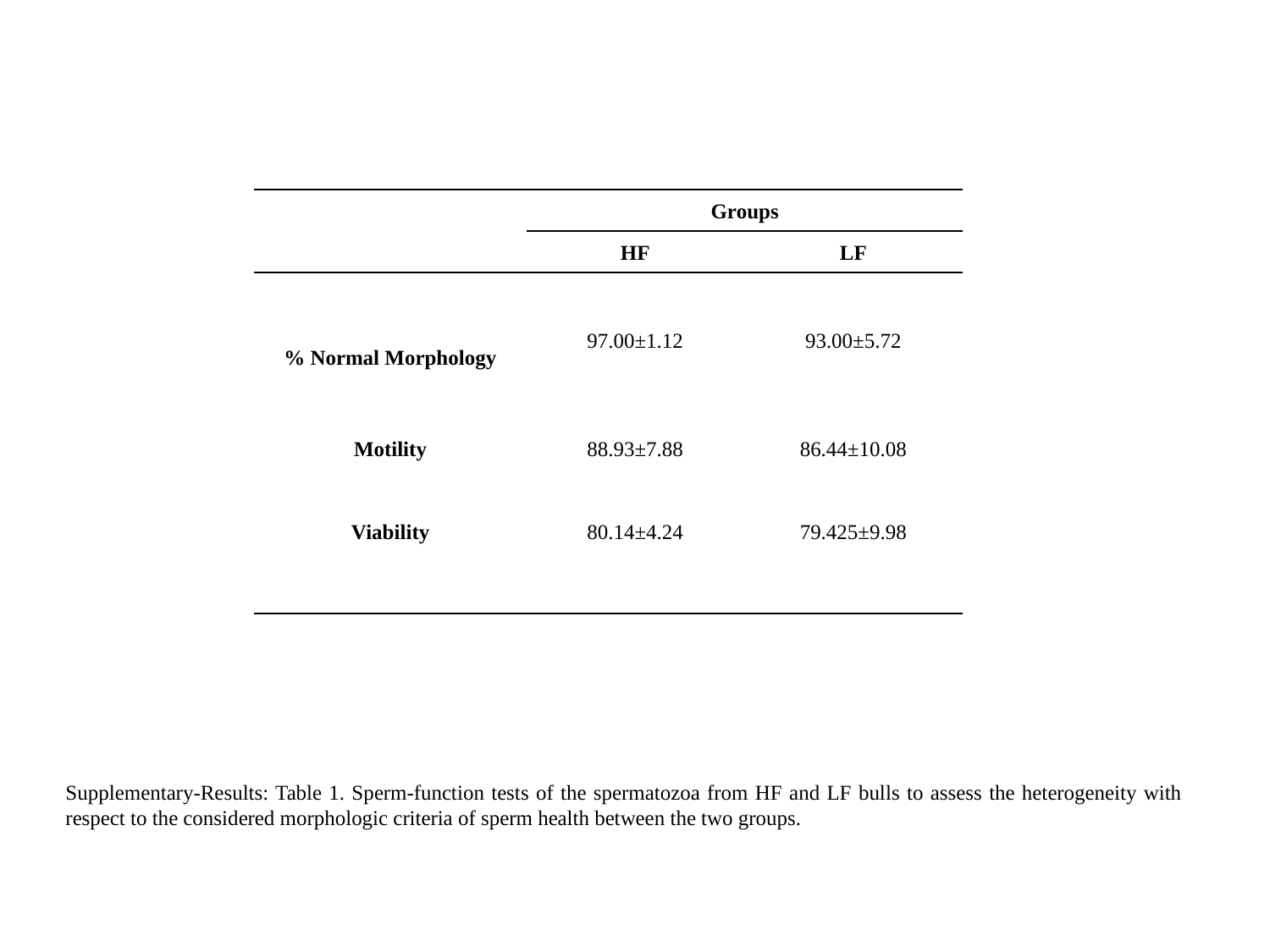

| | | |
| --- | --- | --- |
| | Groups | |
| | HF | LF |
| % Normal Morphology | 97.00±1.12 | 93.00±5.72 |
| Motility | 88.93±7.88 | 86.44±10.08 |
| Viability | 80.14±4.24 | 79.425±9.98 |
| | | |
Supplementary-Results: Table 1. Sperm-function tests of the spermatozoa from HF and LF bulls to assess the heterogeneity with respect to the considered morphologic criteria of sperm health between the two groups.

## Slide 2
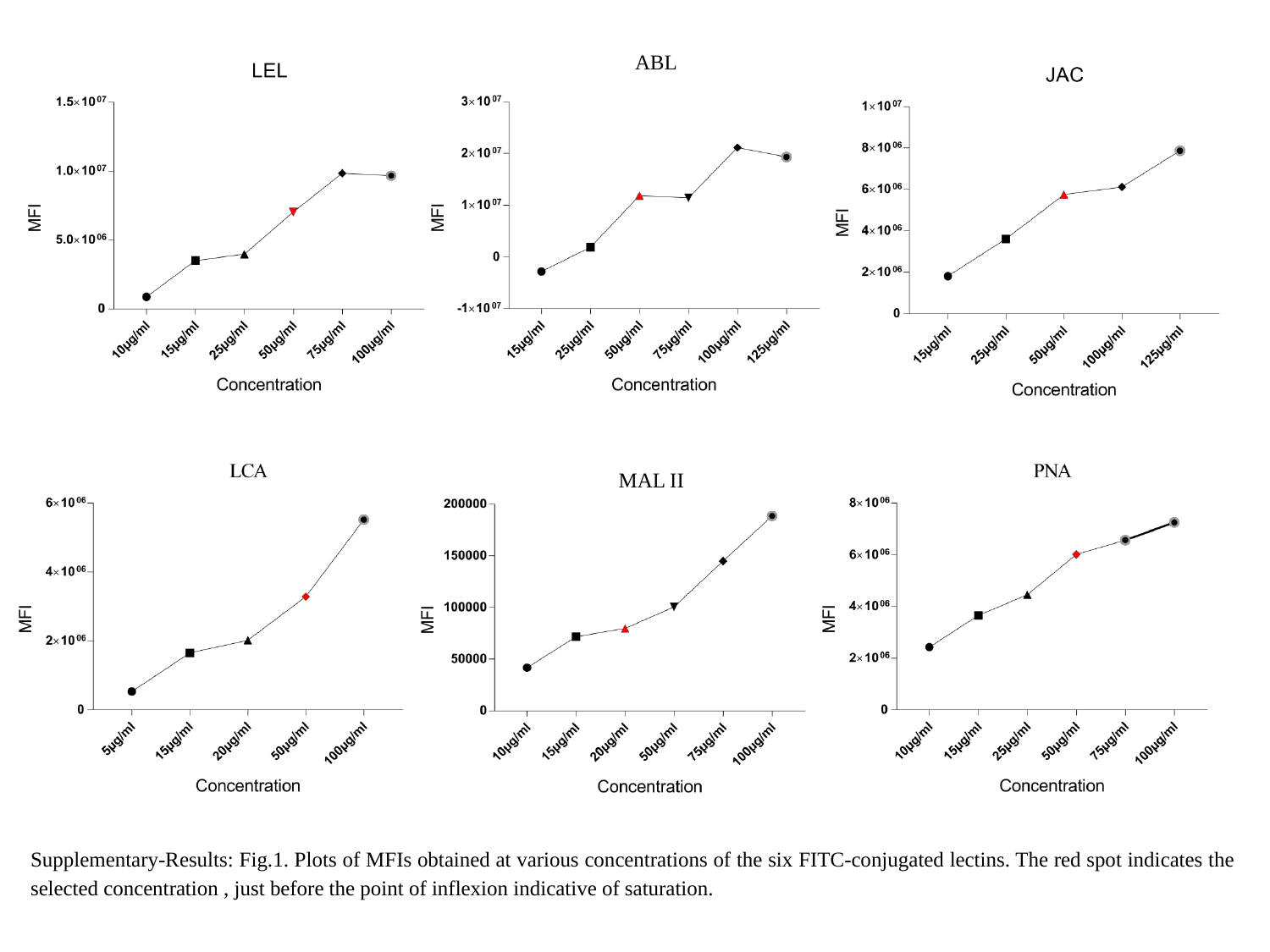

ABL
MAL II
Supplementary-Results: Fig.1. Plots of MFIs obtained at various concentrations of the six FITC-conjugated lectins. The red spot indicates the selected concentration , just before the point of inflexion indicative of saturation.

## Slide 3
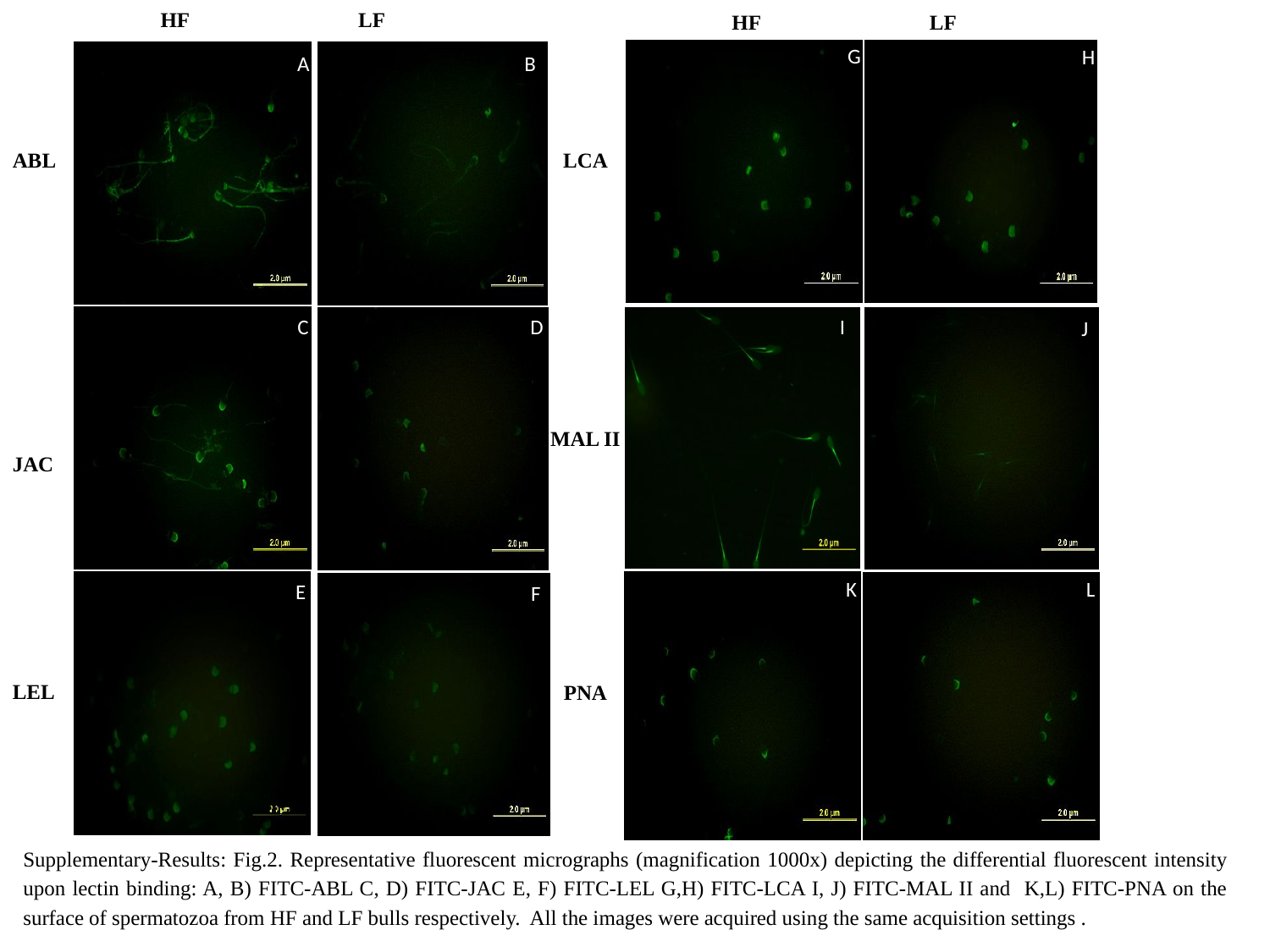

HF LF
HF LF
G
H
A
B
LCA
MAL II
PNA
D
I
C
J
K
L
E
F
ABL
JAC
LEL
Supplementary-Results: Fig.2. Representative fluorescent micrographs (magnification 1000x) depicting the differential fluorescent intensity upon lectin binding: A, B) FITC-ABL C, D) FITC-JAC E, F) FITC-LEL G,H) FITC-LCA I, J) FITC-MAL II and K,L) FITC-PNA on the surface of spermatozoa from HF and LF bulls respectively. All the images were acquired using the same acquisition settings .

## Slide 4
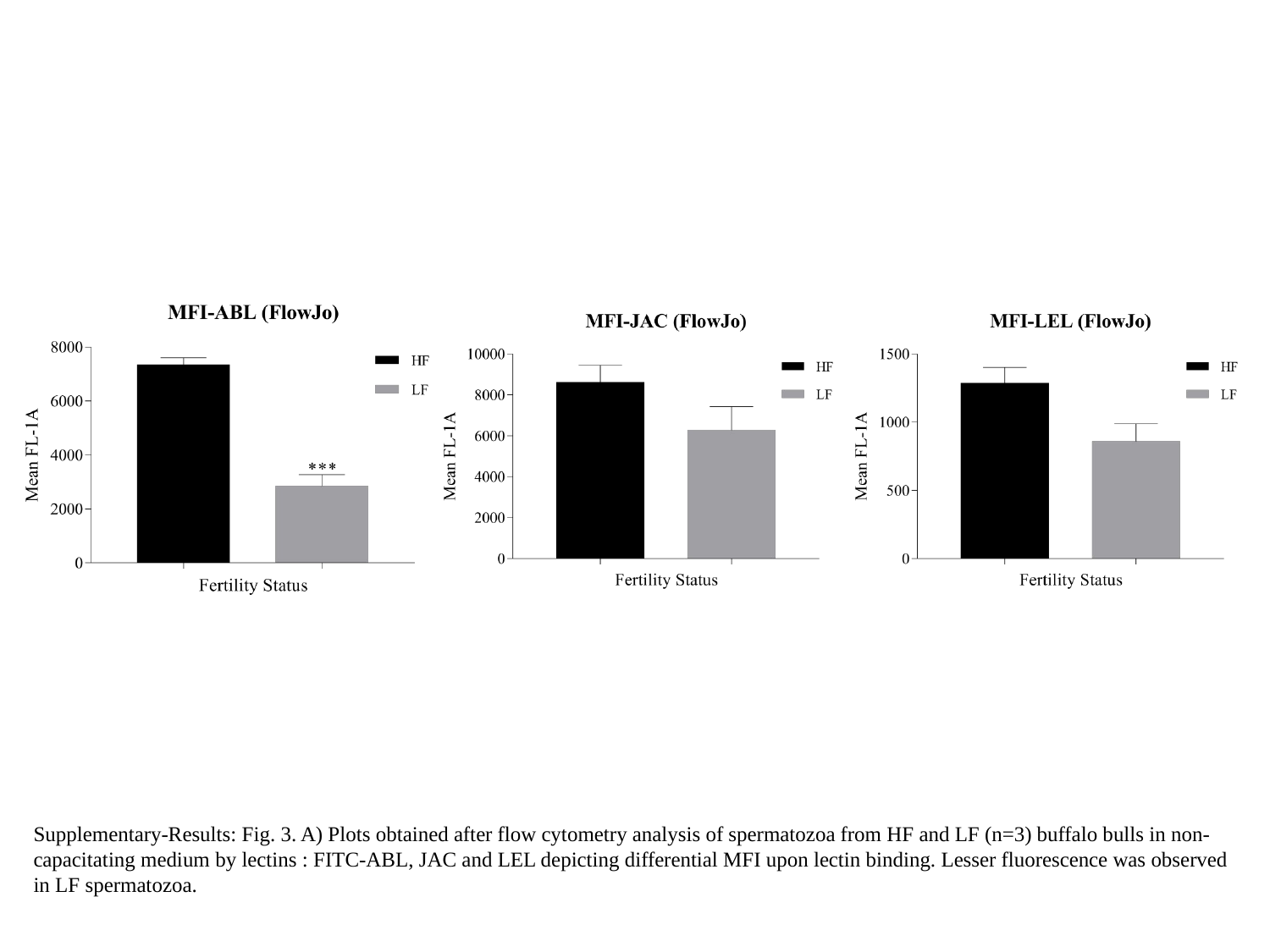

Supplementary-Results: Fig. 3. A) Plots obtained after flow cytometry analysis of spermatozoa from HF and LF (n=3) buffalo bulls in non-capacitating medium by lectins : FITC-ABL, JAC and LEL depicting differential MFI upon lectin binding. Lesser fluorescence was observed in LF spermatozoa.

## Slide 5
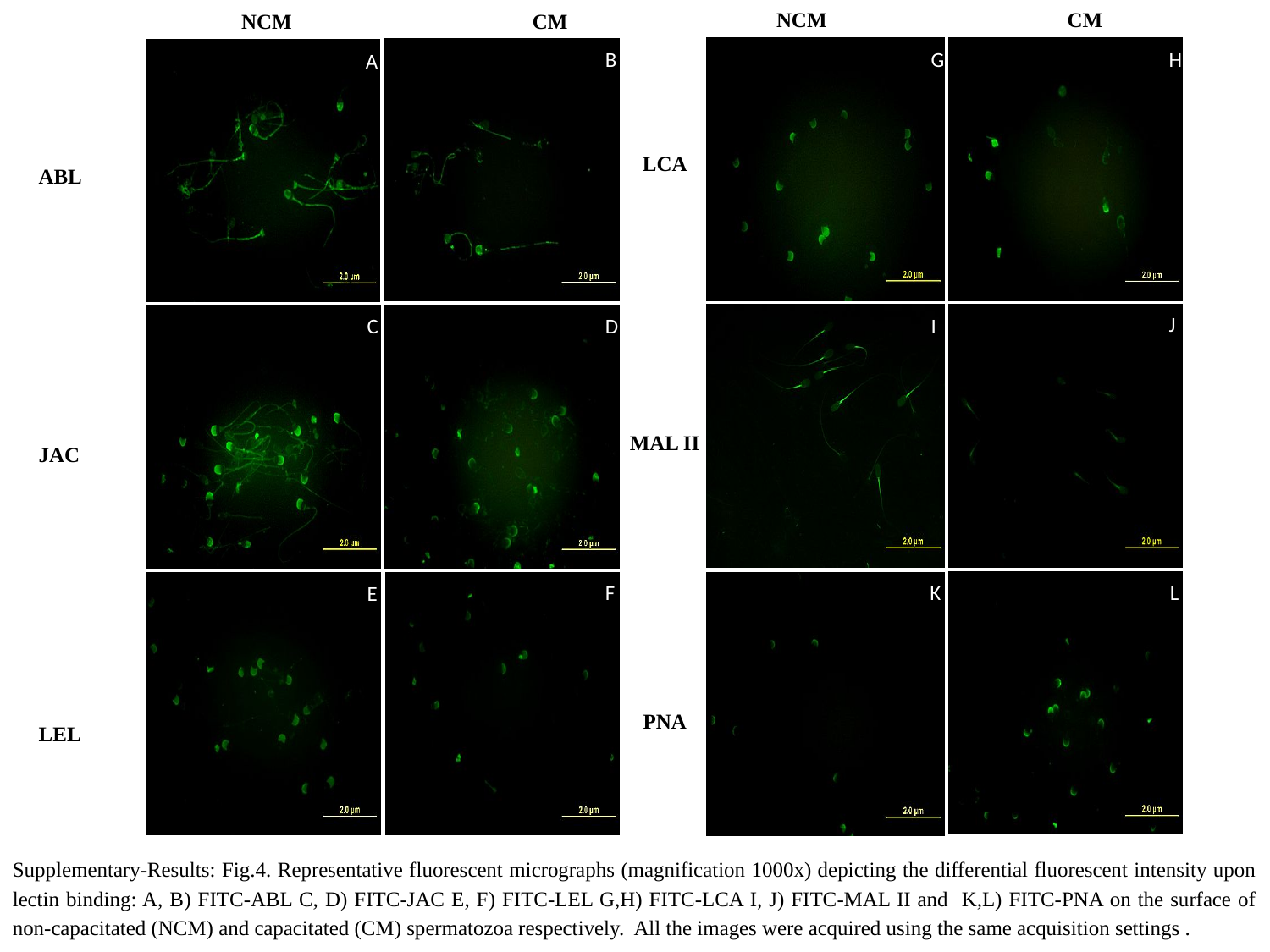

NCM	 CM
NCM	 CM
B
A
C
D
F
E
G
H
J
I
K
L
ABL
JAC
LEL
LCA
MAL II
PNA
Supplementary-Results: Fig.4. Representative fluorescent micrographs (magnification 1000x) depicting the differential fluorescent intensity upon lectin binding: A, B) FITC-ABL C, D) FITC-JAC E, F) FITC-LEL G,H) FITC-LCA I, J) FITC-MAL II and K,L) FITC-PNA on the surface of non-capacitated (NCM) and capacitated (CM) spermatozoa respectively. All the images were acquired using the same acquisition settings .

## Slide 6
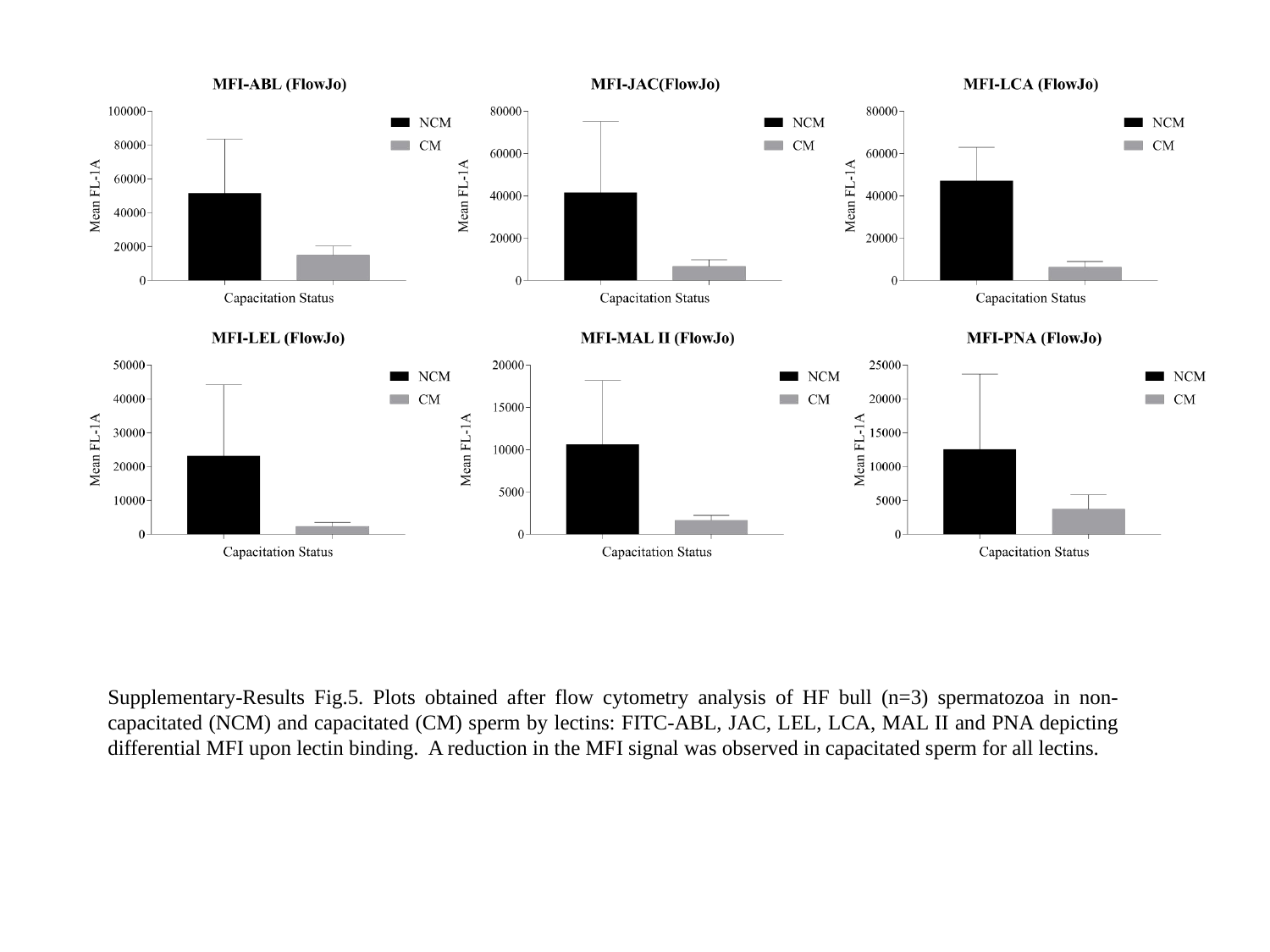

Supplementary-Results Fig.5. Plots obtained after flow cytometry analysis of HF bull (n=3) spermatozoa in non-capacitated (NCM) and capacitated (CM) sperm by lectins: FITC-ABL, JAC, LEL, LCA, MAL II and PNA depicting differential MFI upon lectin binding. A reduction in the MFI signal was observed in capacitated sperm for all lectins.

## Slide 7
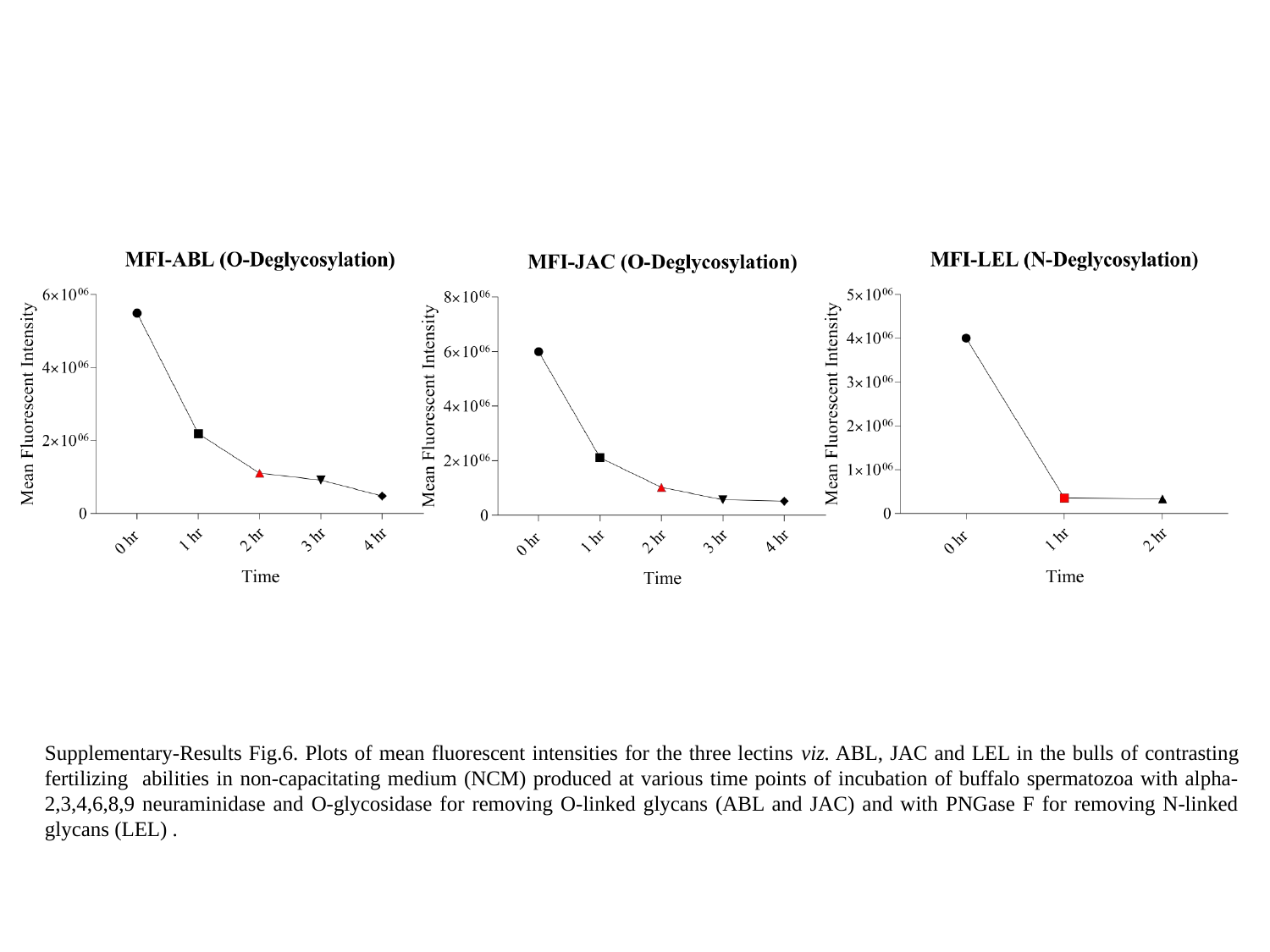

Supplementary-Results Fig.6. Plots of mean fluorescent intensities for the three lectins viz. ABL, JAC and LEL in the bulls of contrasting fertilizing abilities in non-capacitating medium (NCM) produced at various time points of incubation of buffalo spermatozoa with alpha-2,3,4,6,8,9 neuraminidase and O-glycosidase for removing O-linked glycans (ABL and JAC) and with PNGase F for removing N-linked glycans (LEL) .

## Slide 8
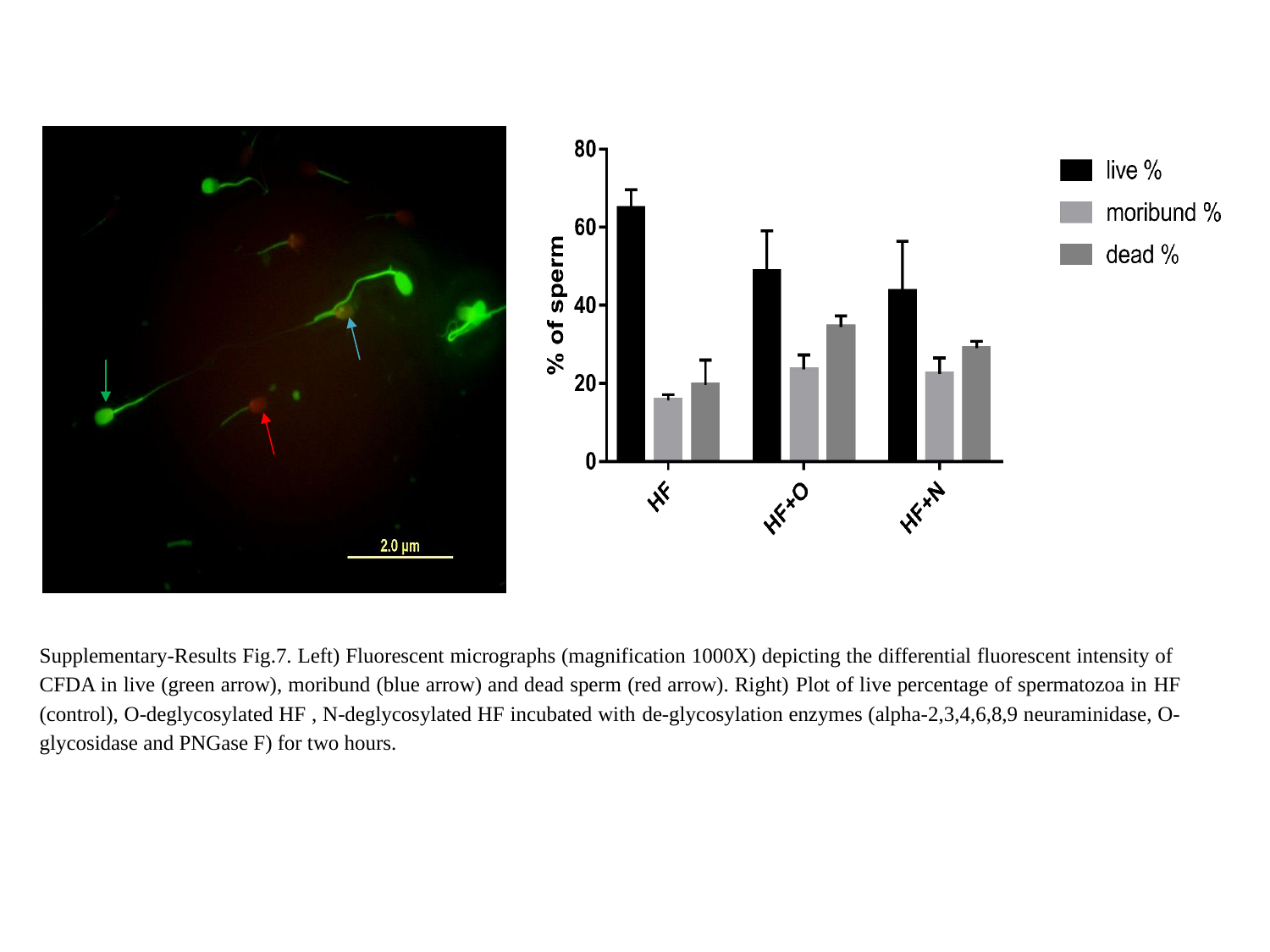

Supplementary-Results Fig.7. Left) Fluorescent micrographs (magnification 1000X) depicting the differential fluorescent intensity of CFDA in live (green arrow), moribund (blue arrow) and dead sperm (red arrow). Right) Plot of live percentage of spermatozoa in HF (control), O-deglycosylated HF , N-deglycosylated HF incubated with de-glycosylation enzymes (alpha-2,3,4,6,8,9 neuraminidase, O-glycosidase and PNGase F) for two hours.

## Slide 9
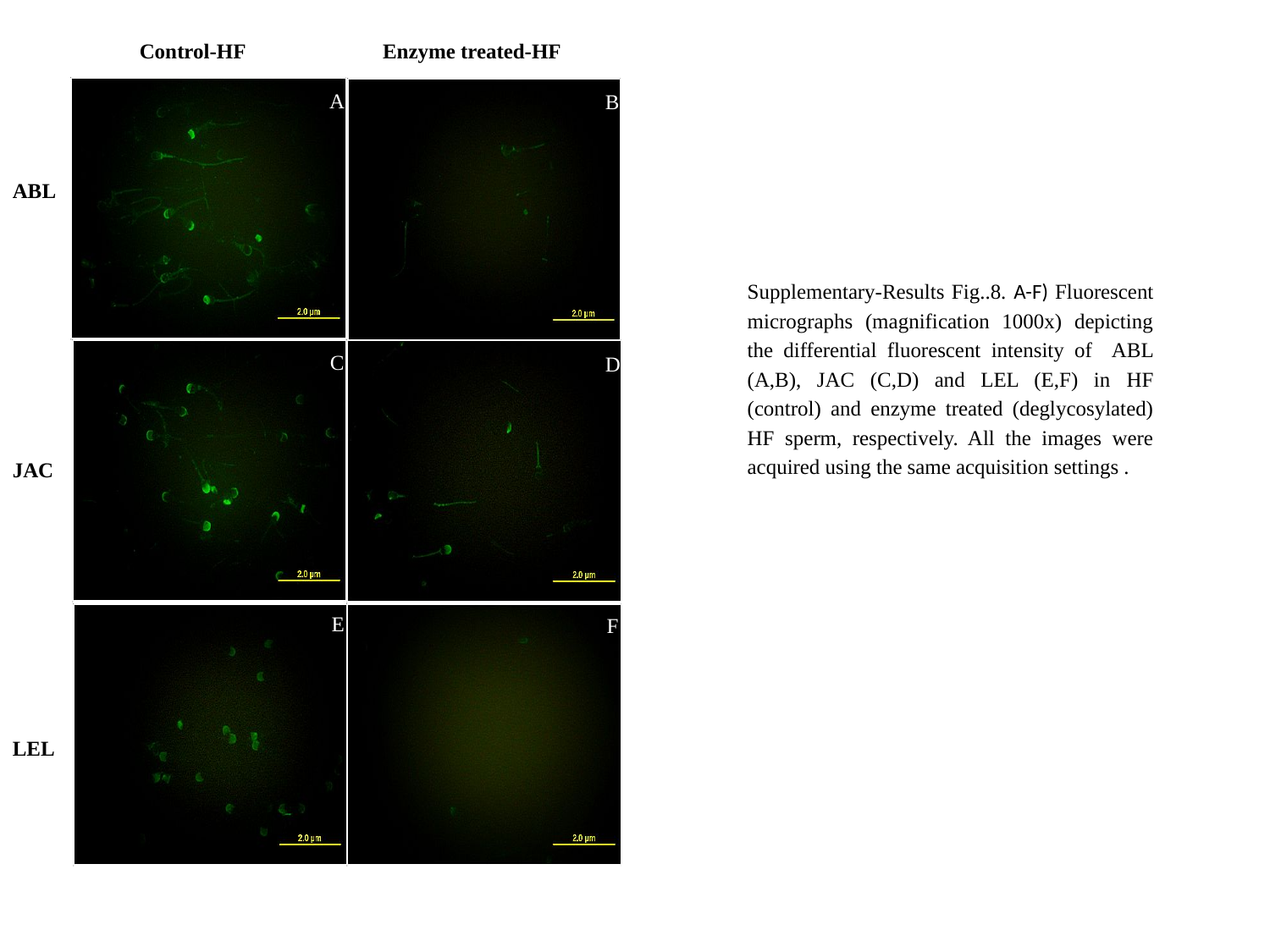

Control-HF Enzyme treated-HF
A
B
ABL
JAC
LEL
C
D
E
F
Supplementary-Results Fig..8. A-F) Fluorescent micrographs (magnification 1000x) depicting the differential fluorescent intensity of ABL (A,B), JAC (C,D) and LEL (E,F) in HF (control) and enzyme treated (deglycosylated) HF sperm, respectively. All the images were acquired using the same acquisition settings .

## Slide 10
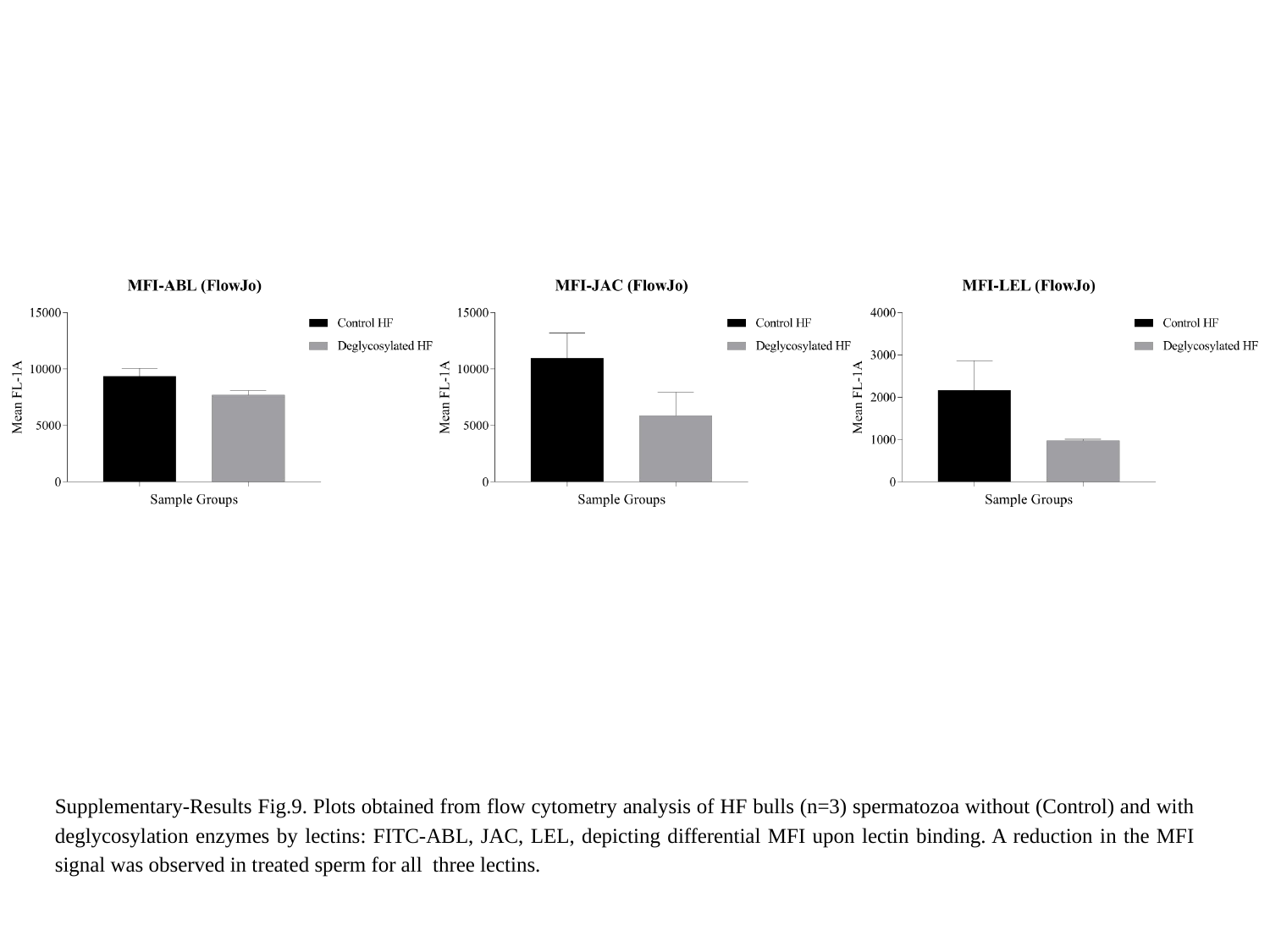

Supplementary-Results Fig.9. Plots obtained from flow cytometry analysis of HF bulls (n=3) spermatozoa without (Control) and with deglycosylation enzymes by lectins: FITC-ABL, JAC, LEL, depicting differential MFI upon lectin binding. A reduction in the MFI signal was observed in treated sperm for all three lectins.

## Slide 11
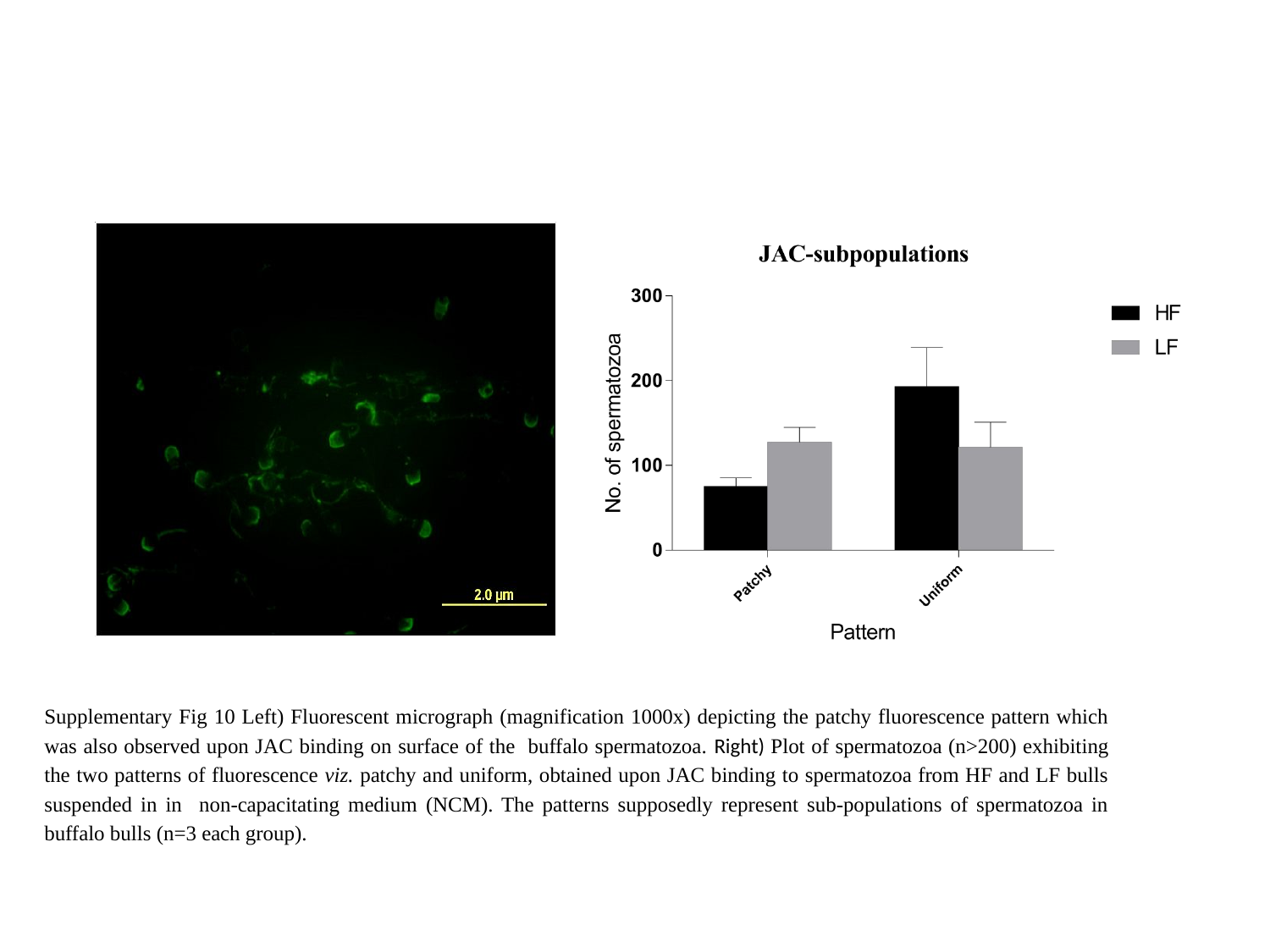

Supplementary Fig 10 Left) Fluorescent micrograph (magnification 1000x) depicting the patchy fluorescence pattern which was also observed upon JAC binding on surface of the buffalo spermatozoa. Right) Plot of spermatozoa (n>200) exhibiting the two patterns of fluorescence viz. patchy and uniform, obtained upon JAC binding to spermatozoa from HF and LF bulls suspended in in non-capacitating medium (NCM). The patterns supposedly represent sub-populations of spermatozoa in buffalo bulls (n=3 each group).
